# Crestal Versus Lateral Window Approaches for Maxillary Sinus Floor Augmentation: A Systematic Review and Meta-Analysis of Randomized Controlled Trials

**DOI:** 10.3390/medicina62101973

**Published:** 2026-10-12

**Authors:** Heera Lee, Sung-Bin Lee, Na Jin Kim, Ji-Eun Lee, Jun-Beom Park

**Affiliations:** 1 Department of Medicine, Graduate School, The Catholic University of Korea, Seoul 06591, Republic of Korea; yysmjj@naver.com (H.L.); sungbin25@naver.com (S.-B.L.); 2 Department of Periodontics, College of Medicine, The Catholic University of Korea, Seoul 06591, Republic of Korea; 3 Medical Library, The Catholic University of Korea, Seoul 06591, Republic of Korea; kimnj@catholic.ac.kr; 4 Department of Periodontology, Korea University Guro Hospital, Seoul 08308, Republic of Korea; 5 Dental Implantology, Graduate School of Clinical Dental Science, The Catholic University of Korea, Seoul 06591, Republic of Korea

**Keywords:** maxillary sinus floor augmentation, crestal approach, lateral window, randomized controlled trials, meta-analysis, GRADE

## Abstract

*Background and Objectives*: Evidence directly comparing crestal and lateral approaches for maxillary sinus floor augmentation remains uncertain. We compared their outcomes and assessed certainty of evidence. *Materials and Methods*: Randomized trials directly comparing both approaches were included. MEDLINE/PubMed, Embase, and the Cochrane Library were searched from inception to 30 July 2026, supplemented by non-systematic Google Scholar searches. Risk of bias and evidence certainty were assessed using RoB 2 and GRADE. Outcomes were pooled as risk ratios (RRs), mean differences (MDs), or standardized mean differences (SMDs), with 95% confidence intervals (CIs). The protocol was registered in PROSPERO (CRD420261447905). *Results*: Thirteen randomized trials (17 reports) were included. No statistically significant differences were found for implant failure (RR 0.50, 95% CI 0.10–2.61), membrane perforation (RR 0.63, 95% CI 0.29–1.38), marginal bone loss at 12 months (MD −0.08 mm, 95% CI −0.31–0.15), vertical bone gain at 12 months (MD −0.81 mm, 95% CI −1.94–0.33) or 6 months (MD −1.07 mm, 95% CI −2.50–0.36), or postoperative pain (SMD −1.56, 95% CI −3.31–0.20). Certainty was low to very low. *Conclusions*: Current evidence does not establish superiority of either approach. Selection should reflect residual bone height, sinus anatomy, treatment objectives, and operator experience.

## 1. Introduction

Maxillary sinus augmentation has become a predictable treatment modality for implant rehabilitation in the atrophic posterior maxilla [1]. Since the lateral window technique was first described by Boyne and James and subsequently modified by Tatum [2,3], various surgical modifications have been introduced to improve treatment outcomes and reduce patient morbidity. More recently, the crestal approach has gained popularity because of its minimally invasive nature and reduced surgical trauma [4,5]. Nevertheless, the selection of the appropriate surgical approach remains dependent on multiple factors, including residual bone height, sinus anatomy, operator experience, and treatment objectives [6,7].

A previous systematic review compared transcrestal and lateral approaches in sites with residual bone height ≤6 mm and found no significant differences in implant survival, Schneiderian membrane perforation, or marginal bone loss [8]. The present review extends this evidence by evaluating a broader set of implant, surgical, radiographic, and patient-reported outcomes with outcome-level RoB 2 assessment and GRADE evaluation. Therefore, this review aimed to compare the two approaches across implant, surgical, radiographic, and patient-reported outcomes and to assess the certainty of the resulting evidence.

## 2. Materials and Methods

### 2.1. Protocol and Eligibility Criteria

This systematic review was conducted and reported in accordance with the PRISMA 2020 statement [9] and was prospectively registered in PROSPERO on 12 July 2026 (CRD420261447905). After completion of the review, the PROSPERO record was amended to reflect the methods actually used. Material deviations from the original registration, together with their rationales and locations in the report, are summarized in Supplementary Table S1. The original registered protocol remains available through the PROSPERO record.

Question: In adults undergoing maxillary sinus floor augmentation with dental implant placement, does the crestal (transalveolar) approach differ from the lateral window approach in implant failure, Schneiderian membrane perforation, peri-implant marginal bone loss, vertical bone gain, or postoperative pain?

Participants: Adults undergoing maxillary sinus floor augmentation with dental implant placement.

Intervention: Crestal (transalveolar) sinus floor augmentation.

Comparison: Lateral window sinus floor augmentation.

Outcomes: Implant failure, Schneiderian membrane perforation, marginal bone loss at 12 months, vertical bone gain at 6 and 12 months, and postoperative pain.

Study design: Randomized and quasi-randomized controlled trials. No quasi-randomized trial met the eligibility criteria; therefore, all included trials were randomized controlled trials.

Studies were eligible if they (1) included adults undergoing maxillary posterior implant placement with sinus floor augmentation, (2) directly compared crestal and lateral approaches, (3) used randomized or quasi-randomized allocation, and (4) reported extractable data for at least one outcome of interest. After full-text assessment, two reports were excluded post hoc because of concerns affecting data interpretability and applicability. This exclusion criterion was not prespecified in the registered protocol. Both reports were independently assessed by HL and JEL, and the exclusion decisions were reached by consensus. Both reports were reintroduced in sensitivity analyses where usable data were available.

### 2.2. Information Sources and Search Strategy

A medical librarian (NJK) developed database-specific search strategies using controlled vocabulary (MeSH in PubMed and the Cochrane Library and Emtree in Embase) and free-text terms. “Crestal approach” and “lateral window,” together with their synonyms, were searched as free-text terms rather than MeSH headings. The search combined a block covering sinus augmentation or endosseous dental implantation with separate crestal/transcrestal and lateral-window blocks using AND; terms within each block were combined using OR. MEDLINE via PubMed, Embase, and the Cochrane Library were searched from inception to 30 July 2026. Eligibility was restricted to English-language reports, as specified in the registered protocol. As a supplementary, non-systematic measure, one reviewer (HL) searched Google Scholar on 30 July 2026 using the free-text query “sinus transcrestal lateral,” with the results limited to publication years from 2025 onward. Search results were reviewed for potentially eligible reports, followed by an additional author-based search for the authors of potentially relevant reports. These searches were not archived, and the number of results reviewed was not recorded; they are therefore reported as supplementary, non-systematic searches rather than as a fully reproducible search strategy. Records identified through this route were processed through the “other methods” pathway of the PRISMA 2020 flow diagram, and two potentially eligible reports were retrieved for full-text assessment. Full database search strategies are provided in Supplementary Table S2.

### 2.3. Study Selection and Data Extraction

Records retrieved from all sources were imported into EndNote 2025 (Clarivate, Philadelphia, PA, USA), and duplicates were removed using the software’s duplicate-detection function with manual verification. No automation tools were used for eligibility screening. Two reviewers (HL and JEL) independently screened titles and abstracts and subsequently assessed potentially eligible full-text reports. Disagreements were resolved by discussion, with SBL acting as an arbiter when required. HL and JEL independently extracted data on study characteristics, numbers randomized and analyzed, intervention and comparator details, unit of analysis, follow-up duration, and the primary outcomes defined for the completed review and additional complications. Study authors were not contacted for additional information, and no study datasets or individual participant data were sought or obtained.

### 2.4. Risk of Bias Assessment

Risk of bias was independently assessed by HL and JEL using the Cochrane RoB 2 tool [10] at the outcome level across five domains: the randomization process, deviations from intended interventions, missing outcome data, measurement of the outcome, and selection of the reported result. Overall judgments were classified as low risk, some concerns, or high risk.

### 2.5. Data Synthesis and Analysis

The meta-analysis was performed using R (Version 4.4.2; R Foundation for Statistical Computing, Vienna, Austria; meta package, version 8.5.0), with the risk ratio (RR) for binary outcomes and the mean difference (MD) or standardized mean difference (SMD, Hedges’ g) for continuous outcomes, together with 95% confidence intervals (CIs), as the summary statistics. Random-effects models using restricted maximum likelihood (REML) were used for all pooled analyses except implant failure, for which a Mantel–Haenszel fixed-effect model was used because events were sparse and no heterogeneity was observed. The Hartung–Knapp adjustment was applied to all continuous-outcome pools. Heterogeneity across studies was assessed using the I^2^ statistic and the chi-square test. For outcomes reported using different analysis units, primary analyses preferentially included patient-, sinus-, or site-level data, whereas implant-level data were examined in sensitivity analyses. This restriction was not applied when it would have left two or fewer trials in a pool. Sensitivity analyses evaluated the inclusion of implant-level data for binary outcomes; exclusion of Xhanari 2019 [11], the within-patient trial, from the pools to which it contributed; addition of the floor-censored Farina [12] data to the marginal-bone-loss analysis; restriction of the vertical-bone-gain analyses to patient- or sinus-level data and exclusion of Mattar 2026 [13]; leave-one-out exclusion of Lv 2022 [14] from the postoperative-pain analysis; and reintroduction of the two reports excluded post hoc where usable data were available. For vertical bone gain at 6 months, the effect of omitting the Hartung–Knapp adjustment was also examined.

Throughout this manuscript, k denotes the number of trials retained in a pooled estimate by the meta package; trials with zero events in both arms are retained in the corresponding forest plot for completeness but are automatically excluded from the pooled estimate by the meta package’s double-zero-event handling, and are reported as contributing no weight rather than as increasing k. In Temmerman 2017 [15], which included two crestal arms (transcrestal sinus floor elevation and implant lift) and one lateral arm, the two crestal arms were combined into a single crestal group before pooling, in accordance with Cochrane Handbook guidance on multi-arm trials [16]; this combined group is labelled “[MERGED]” in the corresponding forest and funnel plots. Xhanari 2019 [11] used a within-patient (split-mouth) design but reported outcomes as two independent groups; it was entered as such, and a sensitivity analysis excluding it was run for both pools to which it contributes. Where a trial reported a median and interquartile range rather than a mean and standard deviation, the mean and standard deviation were estimated using the methods of Wan et al. [17]. For postoperative pain, the earliest reported timepoint within the first 24 h after surgery was used; where a trial reported several timepoints, the day 0–1 value was taken.

### 2.6. Use of Generative Artificial Intelligence

During manuscript preparation, the authors used OpenAI ChatGPT and Codex (OpenAI, San Francisco, CA, USA; https://chatgpt.com/ and https://openai.com/codex/; accessed on 22 September 2026) to assist with language refinement, manuscript organization, methodological clarification, and internal consistency checking across the manuscript and Supplementary Materials. These tools did not independently perform study selection, data extraction, risk-of-bias assessment, GRADE assessment, or statistical analysis. No generative artificial intelligence was used to generate study data or to perform the statistical analyses. For the graphical abstract, an OpenAI image-generation tool was used to create schematic illustrations of the crestal and lateral window approaches, and Codex assisted with the visual layout. All numerical results shown in the graphical abstract were taken from the completed meta-analysis. All AI-assisted suggestions were critically reviewed, verified, and edited by the authors, who take full responsibility for the final content.

### 2.7. Assessment of Reporting Bias

Potential small-study effects arising from reporting bias were assessed using a contour-enhanced funnel plot and Egger’s regression test when at least 10 trials contributed to a pooled analysis. This assessment was performed only for the membrane-perforation all-units sensitivity pool (k = 10); it was not undertaken for the other outcomes because fewer than 10 trials contributed.

### 2.8. Assessment of Certainty of Evidence

Certainty of evidence for each primary outcome was assessed using the GRADE framework [18], considering risk of bias, inconsistency, indirectness, imprecision, and publication bias. Outcome-specific reasons for downgrading are reported in the summary-of-findings table. Downgrading was applied when specific concerns were identified within each domain. In the absence of a validated anchor-based threshold, imprecision for marginal bone loss was assessed using a post hoc distribution-based minimal important difference of approximately 0.30 mm, calculated as half the pooled within-arm standard deviation of the contributing trials.

## 3. Results

### 3.1. Search Results and Study Characteristics

**Figure 1:**
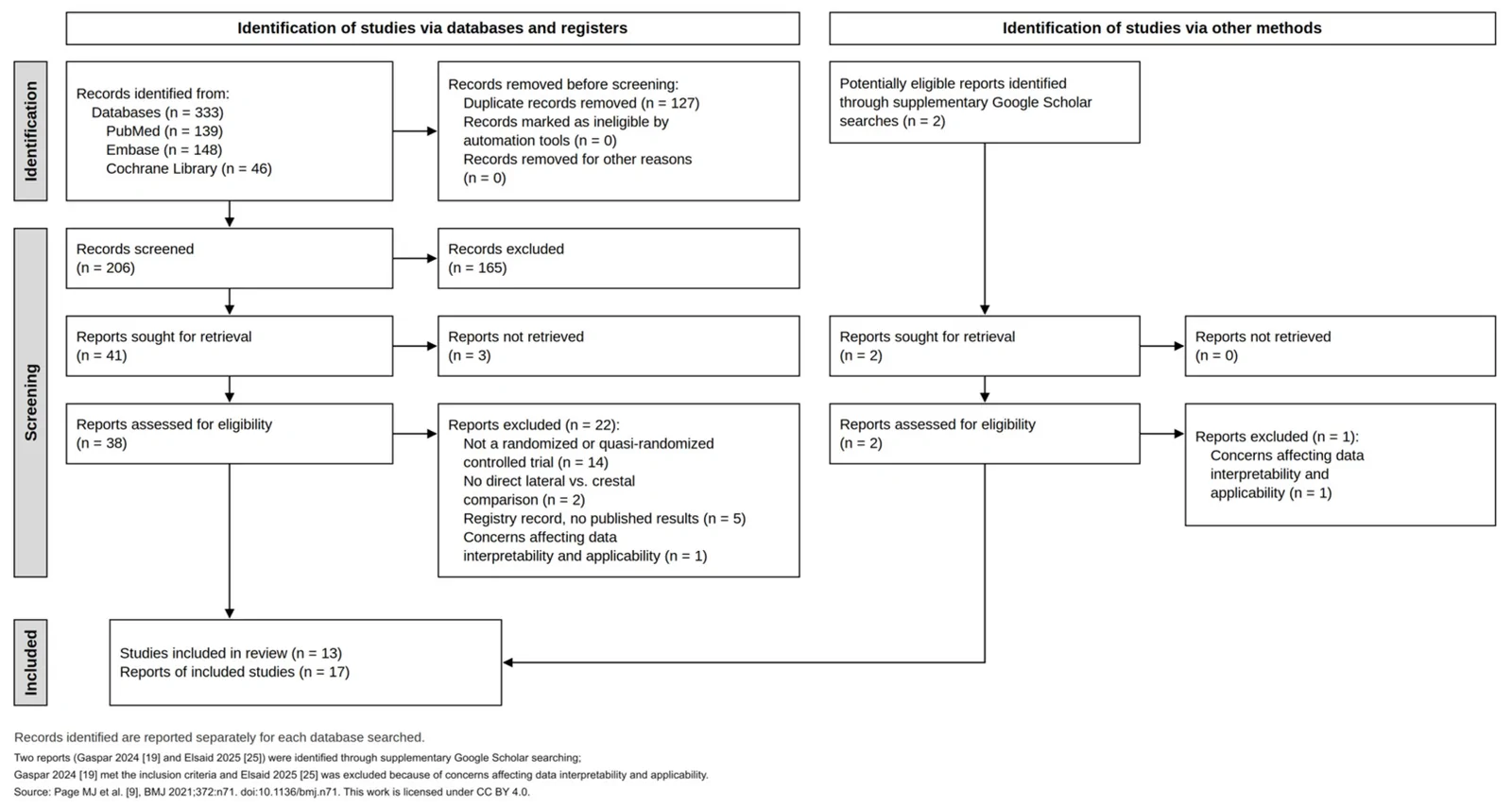
PRISMA 2020 flow diagram of study selection. The diagram shows study identification, screening, eligibility assessment, and inclusion. Reports named in the diagram footnote: Gaspar 2024 [19] and Elsaid 2025 [25]. The flow diagram template is from the PRISMA 2020 statement [9].

Database searches identified 333 records (PubMed, *n* = 139; Embase, *n* = 148; Cochrane Library, *n* = 46). After 127 duplicate database records were removed, 206 database records were screened, of which 165 were excluded based on title and abstract. Forty-one reports were sought for retrieval, of which three could not be retrieved. Of the 38 database-derived reports assessed for eligibility, 22 were excluded (not a randomized or quasi-randomized controlled trial, *n* = 14; no direct lateral versus crestal comparison, *n* = 2; registry record with no published results, *n* = 5; concerns affecting data interpretability and applicability, *n* = 1). Separately, two reports identified through supplementary Google Scholar searching were retrieved and assessed through the “other methods” pathway. Gaspar et al. [19] met the inclusion criteria, whereas the other report was excluded because of substantial internal inconsistencies in the reported results; details are provided in Supplementary Table S3. Ultimately, 13 trials reported in 17 publications were included in the quantitative synthesis; four publications were sequential reports of the Farina trial [12,20,21,22], and two were sequential reports of the Cannizzaro trial [23,24]. Each trial was counted once at the trial level. Figure 1 presents a flowchart of the study selection process, and Supplementary Table S3 lists the reports excluded after full-text assessment, with reasons. The main characteristics of the included studies are shown in Table 1.

### 3.2. Risk of Bias in Included Studies

**Figure 2:**
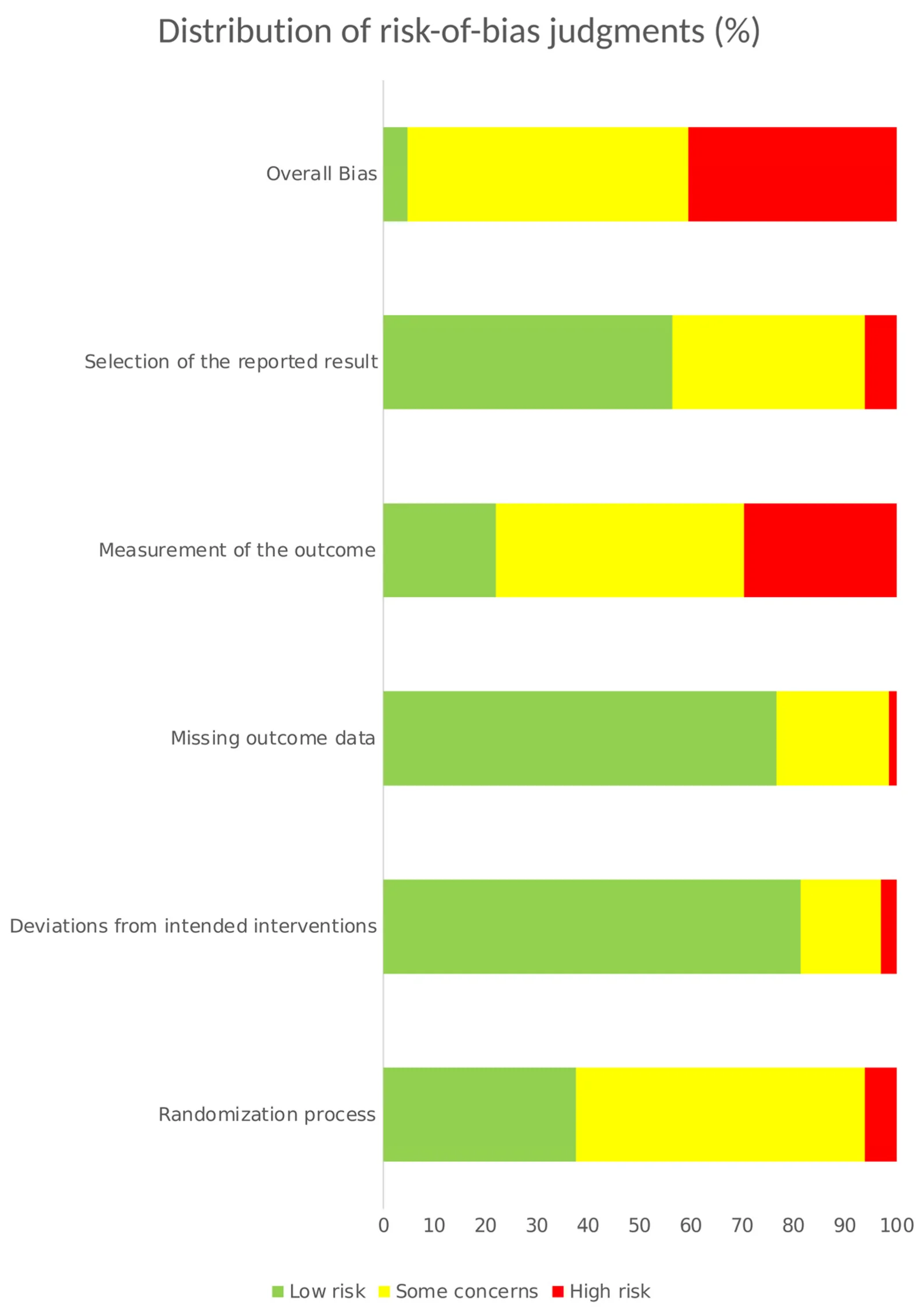
Distribution of RoB 2 judgments across domains and overall. Percentages are based on 64 trial–outcome assessments. Green indicates low risk, yellow some concerns, and red high risk.

Figure 2 presents the distribution of risk-of-bias judgments across the five RoB 2 domains and overall. Following the Cochrane RoB 2 approach, risk of bias was assessed at the level of each outcome contributed by each trial rather than as a single score per trial, yielding 64 study-outcome judgments in total. Across the 64 judgments, 26 (40.6%) were rated overall high risk, 35 (54.7%) some concerns, and 3 (4.7%) low risk; no trial contributed exclusively low-risk judgments across all of its assessed outcomes. High-risk judgments were concentrated among patient-reported pain, discomfort, and satisfaction outcomes, for which all 8 outcome-level judgments were rated high risk. Six were continuous pain-VAS outcomes (Fettouh 2026, Cheng 2026, Gaspar 2024, Farina, Lv 2022, and Temmerman 2017) [4,14,15,19,20,27]. Bacevic 2021 contributed a VAS measure of patient satisfaction, whereas Yu 2017 reported postoperative discomfort as a dichotomous outcome; these two outcomes were reported separately and were not pooled [26,29]. Only 3 judgments in the entire assessment were rated low risk: implant failure in Fettouh 2026 and in Farina, and surgical time in Farina [20,21,27]. A traffic-light plot showing all 64 trial–outcome judgments is provided in Supplementary Figure S1, and full outcome-level RoB 2 judgments for every trial–outcome pair are provided in Supplementary Table S4.

### 3.3. Meta-Analysis

Of the 13 included trials, data were pooled quantitatively for six primary outcomes: implant failure, Schneiderian membrane perforation, marginal bone loss at 12 months, vertical bone gain at 6 and 12 months, and postoperative pain. Effect estimates are expressed as crestal versus lateral (experimental = crestal, control = lateral). A risk ratio below 1 favors the crestal approach, as does a mean difference below 0 for marginal bone loss and a standardized mean difference below 0 for postoperative pain. For vertical bone gain, where a larger value is the desirable outcome, a mean difference below 0 favors the lateral approach. A random-effects model with the Hartung–Knapp adjustment was used for all continuous outcomes. Trials reporting only non-target follow-up intervals (5, 18, or 24 months) were not included in the primary timepoint-specific pools and were described narratively. Exploratory pooling across these disparate timepoints yielded excessive heterogeneity (I^2^ ≈ 99%) and was not used for inference.

### 3.4. Implant Failure

**Figure 3:**
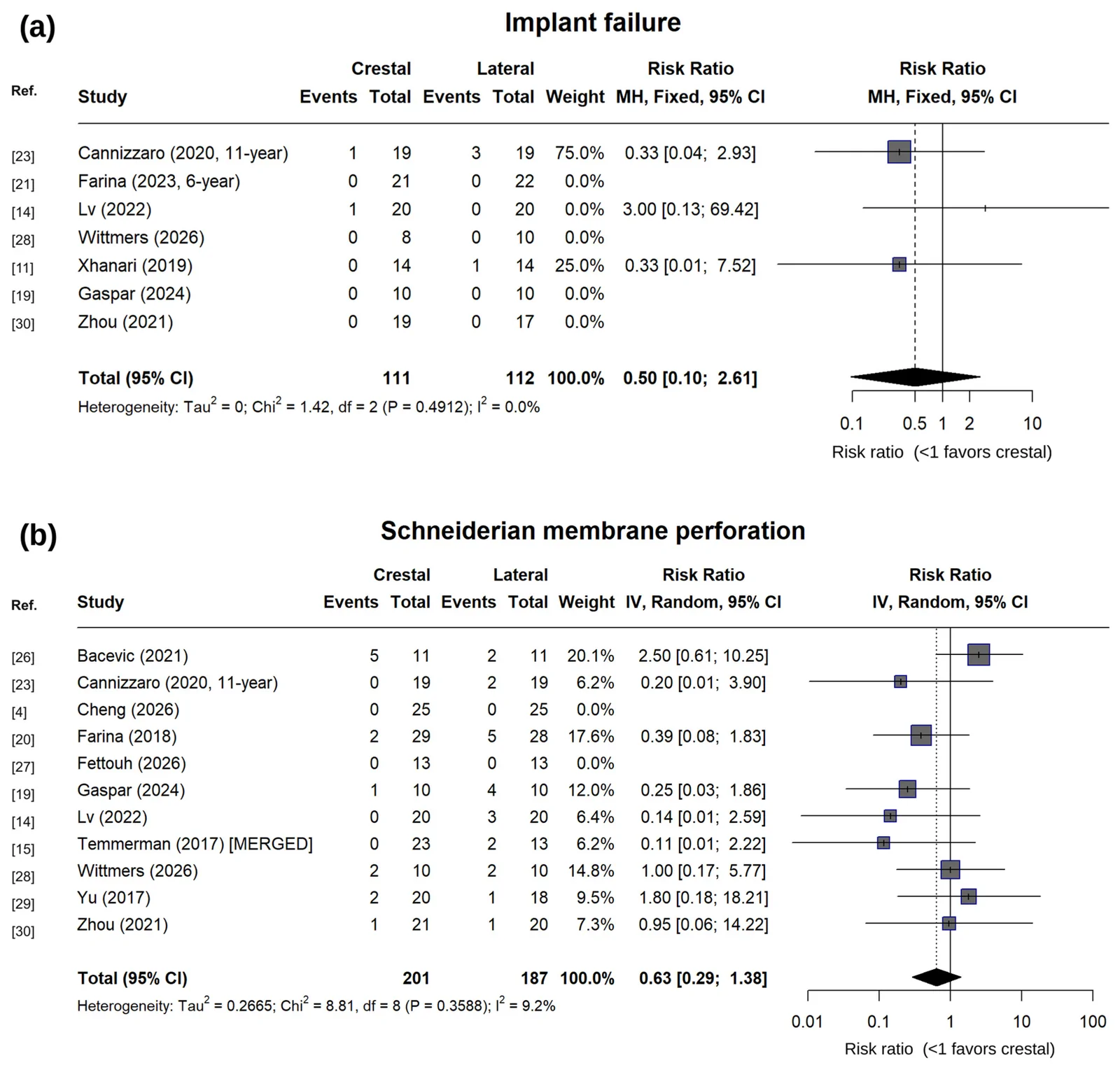
Forest plots of binary outcomes. Forest plots for the binary outcomes comparing the crestal and lateral approaches to maxillary sinus floor augmentation. (**a**) Implant failure (Mantel–Haenszel, fixed effect). (**b**) Schneiderian membrane perforation (inverse variance, random effects). Estimates are risk ratios with 95% confidence intervals, expressed as crestal versus lateral (experimental = crestal, control = lateral); a risk ratio below 1 favors the crestal approach. Trials with zero events in both arms are displayed for completeness but contribute no weight to the pooled estimate. Trials shown: (**a**) Cannizzaro 2020 [23], Farina 2023 [21], Lv 2022 [14], Wittmers 2026 [28], Xhanari 2019 [11], Gaspar 2024 [19], and Zhou 2021 [30]; (**b**) Bacevic 2021 [26], Cannizzaro 2020 [23], Cheng 2026 [4], Farina 2018 [20], Fettouh 2026 [27], Gaspar 2024 [19], Lv 2022 [14], Temmerman 2017 [15], Wittmers 2026 [28], Yu 2017 [29], and Zhou 2021 [30].

Figure 3a shows the forest plot for implant failure. Three trials (Cannizzaro, Xhanari 2019, and Lv 2022) [11,14,23] contributed patient-level data to the primary Mantel–Haenszel analysis. Farina, Wittmers 2026, Gaspar 2024, and Zhou 2021 [19,21,28,30] reported zero events in both arms and were displayed in the forest plot but excluded from the pooled estimate by the double-zero-event rule. Six events occurred among 223 analysis units. The pooled risk ratio was 0.50 (95% CI 0.10–2.61; *p* = 0.41), with no heterogeneity (I^2^ = 0%). A sensitivity analysis additionally including implant-level data from Bacevic 2021, Cheng 2026, Fettouh 2026, Mattar 2026, and Yu 2017 [4,13,26,27,29] (k = 7; Fettouh contributed no weight, reporting zero events in both arms) yielded a similar, non-significant estimate (RR 0.84, 95% CI 0.31–2.29; *p* = 0.74). Implant failure was rare in both arms and did not differ significantly between the crestal and lateral approaches. A sensitivity analysis excluding Xhanari 2019 [11], the only within-patient trial, yielded a similar non-significant estimate (RR 0.67, 95% CI 0.12–3.77; *p* = 0.65; k = 2), without changing the direction or statistical significance of the primary finding. A post hoc sensitivity analysis reintroducing Elsaid 2025 [25] did not change the implant-failure estimate because no implant failures were reported in either arm and the trial therefore contributed no weight.

### 3.5. Membrane Perforation

Figure 3b shows the forest plot for Schneiderian membrane perforation. Nine trials contributed patient-, sinus-, or site-level data (Wittmers 2026, Gaspar 2024, Lv 2022, Zhou 2021, Bacevic 2021, Farina, Yu 2017, Temmerman 2017, and Cannizzaro) [14,15,19,20,23,26,28,29,30]. Eleven trials were displayed in Figure 3b, comprising 388 analysis units and 35 events. Nine trials (312 analysis units; 35 events) contributed to the pooled estimate; Cheng 2026 and Fettouh 2026 [4,27] were excluded from that estimate because both arms had zero events. The pooled RR was 0.63 (95% CI 0.29–1.38; *p* = 0.25), with low heterogeneity (I^2^ = 9.2%). A sensitivity analysis additionally including the implant-level data from Mattar 2026 [13] (k = 10) gave a similar estimate (RR 0.62, 95% CI 0.29–1.30; *p* = 0.21; I^2^ = 0%). Membrane perforation risk did not differ significantly between approaches. A post hoc sensitivity analysis including Bensaha 2011 [31] yielded a similar non-significant estimate (RR 0.54, 95% CI 0.25–1.19; *p* = 0.13; I^2^ = 17.7%; k = 10), without changing the direction of effect.

### 3.6. Marginal Bone Loss

**Figure 4:**
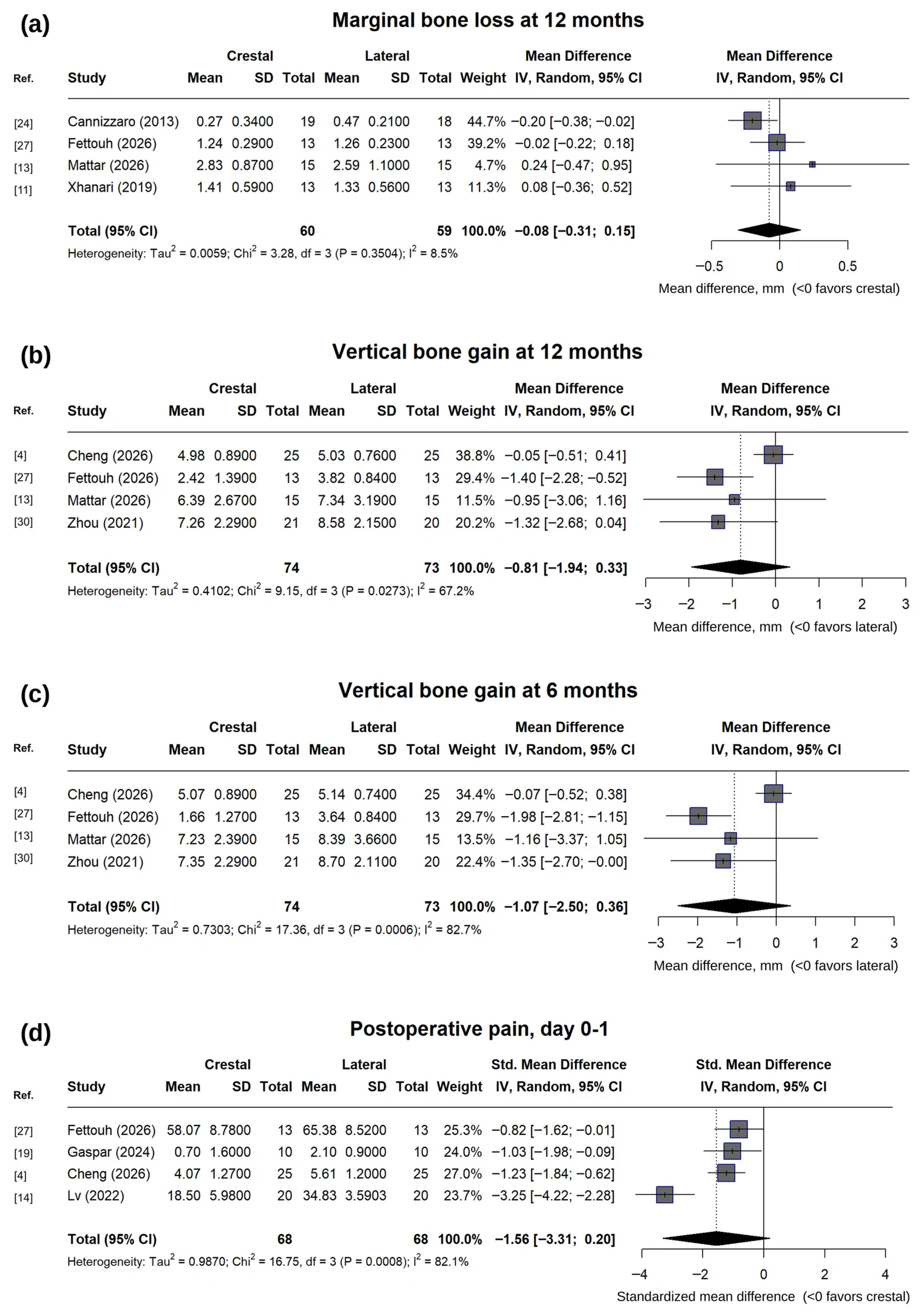
Forest plots of continuous outcomes. Forest plots for the continuous outcomes comparing the crestal and lateral approaches to maxillary sinus floor augmentation. (**a**) Marginal bone loss at 12 months. (**b**) Vertical bone gain at 12 months. (**c**) Vertical bone gain at 6 months. (**d**) Postoperative pain, day 0–1. Estimates are mean differences (**a**–**c**) or standardized mean differences (Hedges’ g; d) with 95% confidence intervals, pooled using a random-effects model (restricted maximum-likelihood estimator) with the Hartung–Knapp adjustment. All estimates are expressed as crestal versus lateral. For marginal bone loss and postoperative pain, a value below zero favors the crestal approach; for vertical bone gain, where a larger value is the desirable outcome, a value below zero favors the lateral approach. Trials shown: (**a**) Cannizzaro 2013 [24], Fettouh 2026 [27], Mattar 2026 [13], and Xhanari 2019 [11]; (**b**,**c**) Cheng 2026 [4], Fettouh 2026 [27], Mattar 2026 [13], and Zhou 2021 [30]; (**d**) Fettouh 2026 [27], Gaspar 2024 [19], Cheng 2026 [4], and Lv 2022 [14].

Figure 4a shows the forest plot for marginal bone loss at 12 months, pooling data from four trials (Cannizzaro, Fettouh 2026, Xhanari 2019, and Mattar 2026 [11,13,24,27]; k = 4). The pooled mean difference was −0.08 mm (95% CI −0.31 to 0.15; *p* = 0.36), with low heterogeneity (I^2^ = 8.5%), indicating no significant difference between approaches. Farina [12] was excluded from the primary pool because the source data were floor-censored at zero and the original articles reported medians analyzed with non-parametric tests rather than means and standard deviations; a sensitivity analysis adding Farina [12] (k = 5) gave a similar, non-significant estimate (MD −0.05 mm, 95% CI −0.19 to 0.10; I^2^ = 9.6%). Excluding Xhanari 2019 [11], the only within-patient trial, likewise yielded a similar non-significant estimate (MD −0.10 mm, 95% CI −0.46 to 0.27; *p* = 0.37; k = 3).

### 3.7. Vertical Bone Gain

Figure 4b,c show the forest plots for vertical bone gain at 12 and 6 months, respectively, each pooling data from four trials (Cheng 2026, Fettouh 2026, Mattar 2026, and Zhou 2021 [4,13,27,30]; k = 4). At 12 months, the pooled MD was −0.81 mm (95% CI −1.94 to 0.33; *p* = 0.11), with substantial heterogeneity (I^2^ = 67.2%). At 6 months, the pooled MD was −1.07 mm (95% CI −2.50 to 0.36; *p* = 0.10), with high heterogeneity (I^2^ = 82.7%). Neither timepoint showed a statistically significant between-group difference. Restricting the analysis to patient-, sinus-, or site-level data would have left only two trials (k = 2), yielding highly imprecise estimates; this analysis is therefore reported below as a sensitivity analysis. Without the Hartung–Knapp adjustment, the 6-month estimate was statistically significant, indicating that this result was sensitive to the choice of statistical method. Sensitivity analyses restricted to patient- or sinus-level data alone (k = 2; Cheng, Zhou) were uninformative (12-month MD −0.52 mm, 95% CI −8.30 to 7.27; 6-month MD −0.55 mm, 95% CI −8.41 to 7.32), and analyses excluding Mattar 2026 [13] (k = 3) gave estimates similar to the primary analysis at both timepoints (I^2^ = 77.6% and 88.3%, respectively). In several pools, mixing of patient-, sinus-, and implant-level data was unavoidable given the small number of available trials, and results should be interpreted with this unit-of-analysis limitation in mind.

### 3.8. Postoperative Pain

Figure 4d shows the forest plot for postoperative pain, pooling data from four trials (Gaspar 2024, Fettouh 2026, Cheng 2026, and Lv 2022 [4,14,19,27]). The pooled SMD was −1.56 (95% CI −3.31 to 0.20; *p* = 0.07), with high heterogeneity (I^2^ = 82.1%), indicating no statistically significant difference between approaches. A leave-one-out sensitivity analysis excluding Lv 2022 substantially reduced heterogeneity and produced a significant result favoring the crestal approach (SMD −1.07, 95% CI −1.61 to −0.53; I^2^ = 0%); this estimate is reported alongside the primary result as a transparency measure rather than as the definitive finding, because no prespecified methodological rule justified excluding Lv 2022 [14] and every trial contributing to this pool was rated overall high risk of bias, which limits confidence in either estimate.

### 3.9. Additional Complications

Additional complications reported across the included trials—including postoperative swelling, bleeding, infection, and prosthetic and biological complications—were not amenable to quantitative pooling beyond the six primary outcomes above because of heterogeneous definitions, timepoints, and units of analysis across studies. These findings are summarized per trial in Table 1 and described narratively below.

Several trials reported greater postoperative pain and swelling after the lateral approach; however, the pooled postoperative pain estimate was not statistically significant, and swelling could not be pooled because of heterogeneous reporting. Other biological and prosthetic complications were heterogeneous in definition and direction across studies and are therefore summarized descriptively in Table 1 rather than pooled quantitatively. No serious adverse event attributable to either approach was reported.

### 3.10. Assessment of Small-Study Effects

**Figure 5:**
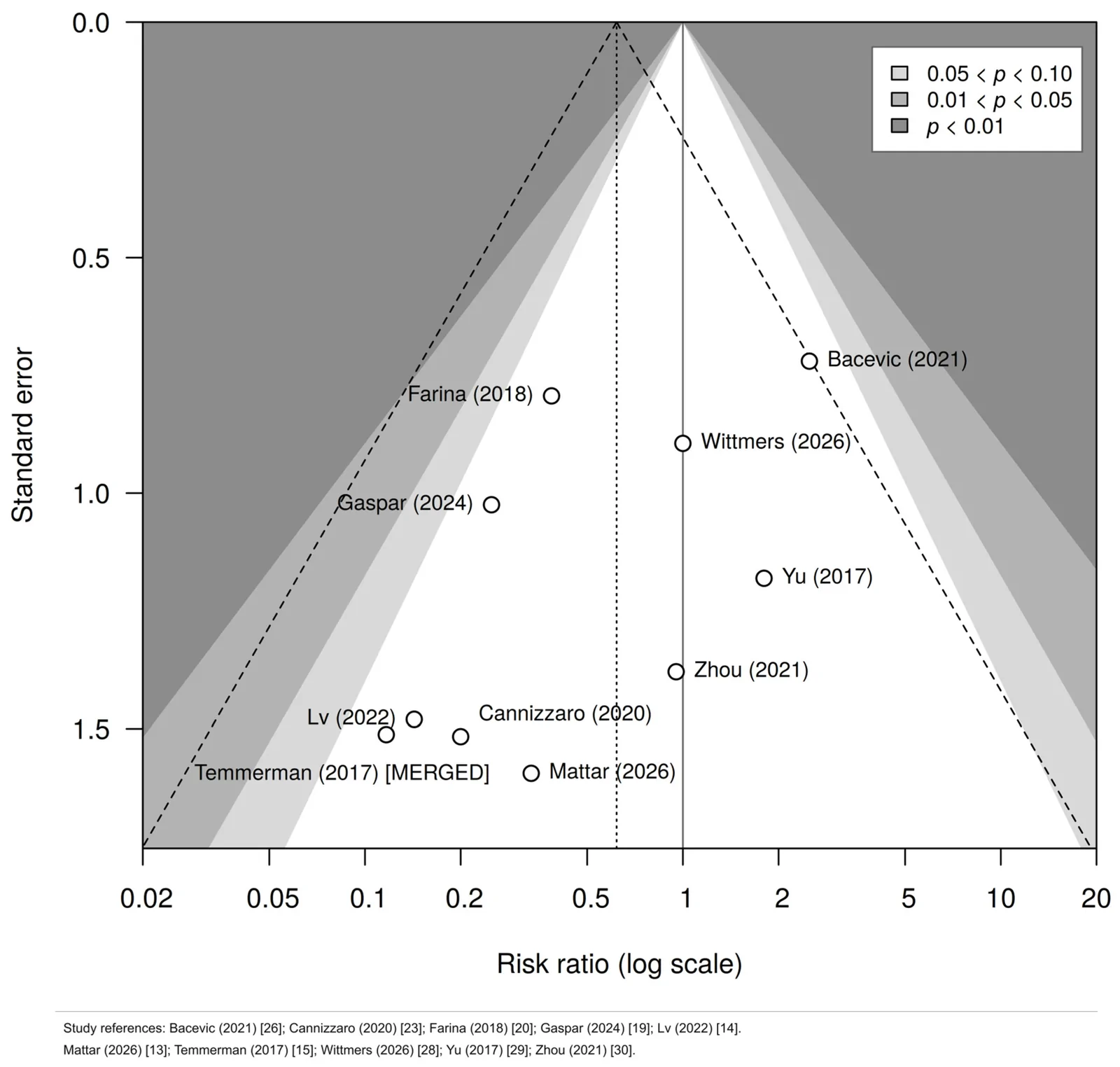
Contour-enhanced funnel plot for membrane perforation. Contour-enhanced funnel plot for the membrane-perforation all-units sensitivity pool (k = 10 trials) used for Egger’s regression test. Shaded regions indicate conventional significance contours (white: *p* > 0.10; light gray: 0.05 < *p* < 0.10; mid gray: 0.01 < *p* < 0.05; dark gray: *p* < 0.01). Funnel plots were not constructed for the other five primary outcomes because fewer than ten trials contributed to each of those pools. Trials shown: Bacevic 2021 [26], Cannizzaro 2020 [23], Farina 2018 [20], Gaspar 2024 [19], Lv 2022 [14], Mattar 2026 [13], Temmerman 2017 [15], Wittmers 2026 [28], Yu 2017 [29], and Zhou 2021 [30].

Funnel-plot asymmetry was assessed only for membrane perforation, the sole outcome with at least 10 trials in the all-units sensitivity pool (k = 10); the primary patient-, sinus-, or site-level pool contained 9 trials. Visual inspection of the contour-enhanced funnel plot (Figure 5) suggested mild asymmetry, and Egger’s regression test did not provide strong evidence of funnel-plot asymmetry (intercept ≈ −1.97, t = −2.05, df = 8, *p* = 0.075); however, with only 10 contributing trials, the test had limited power. Funnel plots and Egger’s tests were not performed for the remaining five outcomes because each pool contained fewer than 10 trials.

### 3.11. Certainty of Evidence

According to the GRADE assessment, the certainty of evidence was low to very low across all six primary outcomes; none reached moderate or high certainty. The full evidence profile, including the reasons for each downgrade, is presented in Table 2. Although all included studies were randomized controlled trials, certainty was downgraded for risk of bias in every pool, reflecting that of the 64 outcome-level risk-of-bias judgments across the 13 trials, 40.6% were rated overall high risk and a further 54.7% some concerns, with only 4.7% rated low risk, and no trial contributed exclusively low-risk judgments across its outcomes. Certainty was further downgraded for imprecision because every pooled confidence interval crossed the line of no effect and several pools comprised as few as three or four trials; for marginal bone loss specifically, imprecision was judged serious because the confidence interval was not fully contained within a distribution-based minimal important difference of approximately 0.30 mm, calculated as half the pooled within-arm standard deviation of the four contributing trials in the absence of a validated anchor-based threshold in the literature. Certainty was additionally downgraded for inconsistency in the vertical bone gain (I^2^ = 67–83%) and postoperative pain (I^2^ = 82%) pools, and particularly for postoperative pain because every trial contributing to that pool was rated overall high risk of bias, yielding very low certainty. Implant failure and marginal bone loss showed low heterogeneity but certainty remained very low for implant failure and low for marginal bone loss because of sparse events, small sample sizes, risk of bias, and, for marginal bone loss, imprecision relative to the minimal important difference.

## 4. Discussion

This meta-analysis found no statistically significant difference between crestal and lateral approaches for any of the six primary outcomes. Point estimates favored the crestal approach for implant failure, membrane perforation, marginal bone loss, and postoperative pain, whereas vertical bone gain favored the lateral approach. However, the low to very low certainty of evidence and wide confidence intervals mean that the absence of statistically significant differences should not be interpreted as evidence of equivalence. Clinically important differences cannot be excluded, particularly for outcomes supported by few trials or events.

The present findings indicate that implant failure was rare and did not differ significantly between the lateral and crestal approaches (RR 0.50, 95% CI 0.10–2.61, k = 3). High implant survival has likewise been reported in systematic reviews of maxillary sinus augmentation using lateral and transalveolar approaches [32,33,34]. Only six implant failures were observed, resulting in a wide confidence interval; therefore, clinically important differences in either direction cannot be excluded.

Although the lateral window approach permits direct visualization of the Schneiderian membrane and placement of larger graft volumes, pooled estimates did not show a statistically significant difference in vertical bone gain at either 6 or 12 months. Point estimates favored the crestal approach for marginal bone loss and the lateral approach for vertical bone gain at both timepoints, but none reached statistical significance, and substantial heterogeneity (I^2^ = 67–83%) in the vertical bone gain pools together with the small number of contributing trials limits confidence in either direction of effect. Sensitivity analyses restricted to patient- or sinus-level data alone were largely uninformative, reflecting the small number of trials reporting outcomes at a unit of analysis matching the unit of randomization. Membrane perforation likewise showed no statistically significant between-group difference (RR 0.63, 95% CI 0.29–1.38); however, the very low certainty of evidence and wide confidence interval preclude a firm conclusion regarding relative risk. The absence of significant differences in implant survival, membrane perforation, and marginal bone loss is broadly consistent with a previous meta-analysis of transcrestal versus lateral sinus floor augmentation in sites with residual bone height ≤ 6 mm [8], although differences in eligibility criteria and analytical methods limit direct comparison.

Postoperative pain also did not differ significantly between approaches (SMD −1.56, 95% CI −3.31 to 0.20). The postoperative pain pool showed high heterogeneity (I^2^ = 82%) and was strongly influenced by a single trial (Lv 2022 [14]) reporting an unusually large effect; a leave-one-out sensitivity analysis excluding this trial produced a significant and far more homogeneous estimate (SMD −1.07, 95% CI −1.61 to −0.53, I^2^ = 0%), which is presented alongside the primary result as a transparency measure rather than as the definitive finding, because no prespecified rule justified excluding Lv 2022 [14] and every trial contributing to this pool was rated overall high risk of bias. Lv 2022 [14] compared a flapless, endoscope-assisted crestal procedure using platelet-rich fibrin with a lateral approach involving full-thickness flap elevation and deproteinized bovine bone mineral. Differences in flap management and grafting material may have contributed to the larger pain contrast. In addition, day-0 pain was reported as medians and interquartile ranges, requiring conversion to means and standard deviations for synthesis. These clinical and reporting characteristics may help explain the different effect estimate, although their contributions cannot be established from the available data.

The observed differences between studies should be interpreted cautiously because substantial clinical heterogeneity was present. Variations in patient selection, residual alveolar bone height, implant placement protocols (simultaneous versus staged), grafting materials, barrier membrane use, follow-up duration, implant systems, and outcome definitions likely contributed to between-study variability. The grafting protocols alone ranged from autogenous bone, deproteinized bovine bone mineral, and allogeneic or alloplastic substitutes to platelet-rich fibrin alone or no graft (Table 1), and growth factor-enhanced grafts such as demineralized dentin matrix combined with recombinant human bone morphogenetic protein-2 have been used, including in lateral sinus grafting, to shorten the healing period before implant loading [35,36,37]. Barrier membranes likewise differ considerably in composition, degradation behavior, and mechanical properties [38], and this may add a further source of clinical heterogeneity. Furthermore, operator experience and differences in surgical techniques may have influenced the reported complication rates. Accordingly, the pooled estimates should be interpreted as averages across diverse clinical settings and should not be assumed to apply uniformly to a particular residual bone height or surgical protocol.

Strengths of this review include prospective protocol registration, duplicate study selection and data extraction, outcome-level RoB 2 assessment, GRADE evaluation, and sensitivity analyses addressing unit-of-analysis concerns and influential studies. The database search strategy required both crestal and lateral terminology to appear in the title, abstract, or keywords. The trial by Gaspar et al. [19] described the crestal intervention as osseodensification and did not use crestal or transcrestal terminology in these fields; it was therefore not retrieved by the database strategy and was identified through supplementary Google Scholar searching. Because the Google Scholar searches were not archived and the number of results reviewed was not recorded, they were not fully reproducible. Other eligible trials using non-standard terminology may consequently have been missed. The within-patient trial by Xhanari et al. [11] was analyzed as reported because paired data required for a within-person analysis were unavailable. Treating the two sides as independent may have affected the precision of the estimates, although exclusion sensitivity analyses did not materially alter the findings. The number of trials contributing to each pooled outcome was small (k = 3–9); of the 64 outcome-level risk-of-bias judgments, only 3 (4.7%) were rated low risk, and no trial contributed exclusively low-risk judgments across its outcomes, which limits the certainty of the pooled estimates. Two reports (Bensaha 2011 [31] and Elsaid 2025 [25]) were excluded post hoc because of concerns affecting data interpretability and applicability; this deviation from the registered protocol is disclosed here. Both reports were independently assessed by two reviewers, and the exclusion decisions were reached by consensus. Returning Bensaha 2011 [31] to the membrane-perforation pool changed the estimate from RR 0.63 (95% CI 0.29–1.38; I^2^ = 9.2%) to RR 0.54 (95% CI 0.25–1.19; *p* = 0.13; I^2^ = 17.7%; k = 10), without changing the direction or statistical significance of the finding; Elsaid 2025 [25] reported no implant failures in either arm and therefore carried no weight. Neither exclusion alters any conclusion of this review. The minimal important difference used to judge the precision of the marginal bone loss estimate (≈0.30 mm) was derived from this review’s own pooled data after the confidence interval was already known, rather than from a prespecified, externally validated anchor; this post hoc, data-dependent choice is disclosed as a methodological limitation. Differences in follow-up duration and outcome assessment limited direct comparison across studies, and the included studies evaluated a wide range of grafting materials and implant systems, introducing additional heterogeneity. For several outcomes—particularly vertical bone gain and postoperative pain—a small number of trials and mixed units of analysis further reduced the precision of the pooled estimates. These limitations reduce the certainty of the pooled estimates and should be considered when interpreting the findings. The restriction to English-language reports may also have introduced language bias. Small-study effects could not be formally assessed for five of the six primary outcomes because fewer than 10 trials contributed to each analysis.

Future research should focus on well-designed multicenter randomized controlled trials directly comparing lateral and crestal approaches using standardized definitions of implant success, complication reporting, and radiographic outcome assessment. Longer follow-up periods are also required to evaluate long-term implant survival, graft stability, patient-reported outcomes, and cost-effectiveness.

## 5. Conclusions

In this meta-analysis of 13 randomized controlled trials, no statistically significant differences were detected between crestal and lateral approaches for implant failure, Schneiderian membrane perforation, marginal bone loss, vertical bone gain, or postoperative pain. However, the certainty of evidence was low to very low, and wide confidence intervals preclude definitive conclusions of equivalence or superiority. Until more robust evidence is available, selection of the surgical approach may be guided by residual bone height, sinus anatomy, treatment objectives, and operator experience. Larger trials with standardized outcome definitions and consistent units of analysis are required.

## Figures and Tables

**Table 1:** Main characteristics of the 13 included trials (17 reports).

Study Author (Year)	Study Design	Sample Size (Crestal: Lateral) and Unit	Test Procedure	Control Procedure	Results	Follow-Up
Bacevic (2021) [26]	Randomized controlled trial	Crestal 11: Lateral 11 (22 sinuses/22 patients)	Crestal hydraulic thread antral system (HTA), Bio-Oss collagen, simultaneous implant	Lateral window, Bio-Oss collagen, simultaneous implant	Implant survival: 14/16 crestal (including HTA→LW conversions) vs. 12/12 lateral at 1 y. Qualitative radiographic assessment showed adequate augmentation in both groups. HTA → LW conversion: 3/11; membrane perforation: 5/11 HTA vs. 2/11 LW. Postoperative morbidity was higher with LW	1 week, 6 months, 1 year
Cannizzaro (2013) [24]	Randomized controlled trial	Crestal (short) 20: Lateral (long) 20 (40 sinuses)	Crestal mini-sinus lift (Cosci technique), 8 mm implant, autogenous graft	Lateral window, 10–16 mm implant, 50% Bio-Oss/50% autogenous graft	Implant-level survival 19/20 (short) vs. 15/20 (long) at 5 y; the pooled analysis uses the patient-level failure counts reported at 11 years (1/19 vs. 3/19) Marginal bone loss: 0.41 mm (short) vs. 0.72 mm (long), *p* = 0.028 4/20 vs. 8/20 patients with ≥1 complication (not significant); abscess/sinusitis in long-implant group led to 4 lost implants	Baseline, 45 d, 1 y, 5 y
Cannizzaro (2020) [23]	Randomized controlled trial	Crestal (short) 19: Lateral (long) 19 analyzed at 11 y	(as above)	(as above)	Survival unchanged from 5-y report (no new failures 5–11 y) Marginal bone loss continued to accrue in both groups; no between-group difference by 11 y Cumulative complications increased 5 → 11 y in both groups (mucositis, prosthetic issues)	Baseline, 45 d, 1, 5, 11 y
Cheng (2026) [4]	Randomized controlled trial	Crestal 25: Lateral 25 (50 sinuses)	Modified crestal approach, simultaneous implant	Lateral window sinus floor elevation, simultaneous implant	Survival 25/25 (crestal) vs. 24/25 (lateral), 1 y Gained bone height comparable between groups at 1 y No membrane perforation in either group; mucositis 2/25 (crestal) vs. 3/25 (lateral)	Baseline, immediate post-op, 3, 6, 12 months
Farina (2018) [20]	Randomized controlled trial	Crestal (tSFE) 29: Lateral (lSFE) 28 (57 patients)	Transcrestal SFE with DBBM and a collagen matrix, simultaneous implant placement	Lateral window SFE with DBBM and a collagen membrane, simultaneous implant placement	No implants lost at 6-month loading (2 early tSFE failures pre-loading) Not assessed at this timepoint Pain was significantly higher with the crestal approach on the day of surgery, with no significant between-group difference from days 1 to 14; swelling was less frequent with the crestal approach; membrane perforation reported in both groups	Day 0, 1, 2, 3, 4, 7, 14; 6 months (loading)
Farina (2019) [12]	Companion report—12-month radiographic outcomes	Crestal 26: Lateral 28 (per-protocol analyzed implants)	(as above)	(as above)	Proportion of the implant surface in contact with the peri-implant radiopaque area assessed at 12 months Marginal bone loss (mesial/distal) at 6 and 12 months Not the focus of this report	Baseline, 6, 12 months
Farina (2022) [22]	Companion report—3-year follow-up (per-protocol)	Crestal 21: Lateral 24 (per-protocol analyzed implants)	(as above)	(as above)	Implant survival 21/21 vs. 24/24 at 3 y (per-protocol) Proportion of the implant surface in contact with the peri-implant radiopaque area and marginal bone level remained stable from 1 to 3 years, with no significant between-group difference Peri-implant mucositis/peri-implantitis prevalence was similar between groups	Baseline, 6 mo, 1 y, 3 y
Farina (2023) [21]	Companion report—6-year follow-up	Crestal 21: Lateral 22 (analyzed at 6 y)	(as above)	(as above)	Six-year completers: 0/21 tSFE vs. 0/22 lSFE failures; early failures excluded. Marginal bone level change 6 y vs. 1 y baseline, no significant between-group difference Peri-implant disease prevalence assessed at 6 y	Baseline, 6 mo, 1, 3, 6 y
Fettouh (2026) [27]	Randomized controlled trial	Crestal 13: Lateral 13 (26 patients)	Crestal implant approach, implant apex tents membrane, no graft	Graftless lateral window, membrane tenting by implant	Survival 13/13 vs. 13/13, 12 months Vertical bone gain significantly greater with lateral approach (12 mo) Marginal bone loss comparable; postoperative pain/swelling significantly lower with crestal approach	T0 (placement), 6, 12 months
Gaspar (2024) [19]	Randomized controlled trial	Crestal 10: Lateral 10 (20 patients)	Crestal sinus lift, simultaneous 10 mm implant	Lateral window sinus lift, simultaneous 10 mm implant	Osseointegration success 10/10 vs. 10/10 at 1 y ISQ increased over time in both groups, no between-group difference at 6 months Postoperative pain and OHIP-14 scores were significantly lower with crestal approach over the first postoperative week	Day 0–7 (daily), 6 months, 1 year
Lv (2022) [14]	Randomized controlled trial	Crestal (PESS) 20: Lateral (LSFE) 20 (40 patients)	Flapless endoscope-assisted osteotome SFE with platelet-rich fibrin, simultaneous implant	Lateral window SFE with deproteinized bovine bone mineral, simultaneous implant	18-month implant survival: 25/26 PESS vs. 31/31 LSFE; patient-level failure: 1/20 vs. 0/20 Radiographic bone height gain and graft maturation (SGRI) assessed at 3, 6, 18 months Swelling markedly higher with lateral approach on postoperative days 0–7; membrane perforation 0/20 vs. 3/20	T0, 3, 6, 18 months
Mattar (2026) [13]	Randomized controlled trial	Crestal 15: Lateral 15 (30 sinuses)	Crestal sinus elevation (osteotome), simultaneous implant, allogeneic graft	Open (lateral) sinus elevation, simultaneous implant, allogeneic graft	Implant success comparable between groups (3 vs. 2 failures) ISQ, plaque index and radiographic bone parameters not significantly different at 12 months Crestal approach: lower bleeding index and less buccal bone loss; more favorable patient-reported edema	T0–T4 (0, immediate, 6, 9, 12 months)
Temmerman (2017) [15]	Randomized controlled trial (single-center pilot)	Transcrestal (tSFE) 8 + Implant-lift (IL) 15 sites: Lateral 13 sites	Transcrestal SFE (osteotome) or implant-lift SFE, ±simultaneous implant	Lateral window SFE, ±simultaneous implant	Not reported (pilot study; no formal survival analysis) Graft/membrane volume by CBCT at 1 and 6 weeks Membrane perforation observed in lateral group; postoperative pain/swelling higher with lateral approach over 7 days	T0, 1 week (T1), 6 weeks (T2)
Wittmers (2026) [28]	Randomized controlled trial (interim report of an ongoing trial)	Crestal (tSFE) 10: Lateral (lSFE) 10 (20 patients, planned 74)	Transcrestal hydrodynamic SFE (Jeder system), staged implant	Conventional lateral window SFE, staged implant	Survival 8/8 vs. 10/10 at 1 year (post hoc, after early discontinuation) Subantral bone height gain significantly greater with lateral approach at 5 months All tSFE sites required re-augmentation to place a ≥10 mm implant, vs. 3/10 lSFE sites	Intraoperative, 5 months (CBCT/biopsy), 1 year (loaded implants)
Xhanari (2019) [11]	Randomized controlled trial	Crestal 15: Lateral 15 (bilateral sites in 15 patients)	Crestal sinus lift, simultaneous implant	Lateral window sinus lift, simultaneous implant	Implant failure 0/14 (crestal) vs. 1/14 (lateral), 1 year Marginal bone level change comparable between sides at 1 year Patient preference strongly favored the crestal side at every timepoint (0 patients ever preferred lateral)	6 months (loading); 1 year after loading
Yu (2017) [29]	Randomized controlled trial	Crestal (short implants) 20: Lateral (long implants) 18 (38 patients)	Short (6.5 mm) implant with crestal SFE, no graft	Long implant (11 or 12.5 mm) with lateral window SFE	Implant failure: 0/38 short vs. 1/41 long implants at 2 y. Marginal bone loss was comparable; endosinus bone gain was greater with lateral SFE (2.94 vs. 10.19 mm, crestal vs. lateral). Postoperative discomfort: 5/20 short vs. 15/18 long-implant patients.	Baseline, 3-month visits, 12, 24 months
Zhou (2021) [30]	Randomized controlled trial	Crestal (TSFE) 19: Lateral (LSFE) 17 (36 patients)	Transcrestal SFE, simultaneous implant	Lateral window SFE, simultaneous implant	Implant success 19/19 (crestal) vs. 17/17 (lateral), 24 months ESBG and ABH did not differ significantly immediately after surgery; the lateral group showed significantly higher values during subsequent follow-up. Small perforations: 1/21 TSFE vs. 1/20 LSFE sites; neither compromised surgery.	Baseline, 6, 12, 18, 24 months

Four reports (Farina 2018, 2019, 2022, 2023) [12,20,21,22] are sequential follow-ups of a single trial, and two (Cannizzaro 2013, 2020) [23,24] are sequential follow-ups of another; each trial is counted once in the synthesis. Cheng 2026 [4] is titled as a prospective study but reports random allocation of participants to the two approaches, and was therefore classified as a randomized controlled trial. Sample sizes are the numbers randomized for primary trial reports; for sequential follow-up reports, the numbers analyzed at the reported follow-up are shown where applicable. Numbers contributing to individual meta-analyses may differ because of loss to follow-up or because outcomes were reported at the patient, sinus, site, or implant level. Abbreviations: ABH, apical implant bone height; CBCT, cone-beam computed tomography; DBBM, deproteinized bovine bone mineral; ESBG, endo-sinus bone gain; HTA, hydraulic thread antral system; IL, implant lift; ISQ, implant stability quotient; LSFE/lSFE, lateral sinus floor elevation; LW, lateral window; OHIP-14, Oral Health Impact Profile-14; PESS, flapless endoscope-assisted osteotome sinus floor elevation with platelet-rich fibrin (terminology used by Lv et al.); SFE, sinus floor elevation; SGRI, sinus grafting remodelling index; TSFE/tSFE, transcrestal sinus floor elevation.

**Table 2:** Summary of findings for crestal versus lateral sinus augmentation.

Outcome (Timepoint)	No. of Analysis Units (Trials)	Relative Effect or Mean Difference (95% CI)	Anticipated Absolute Effects	Certainty of the Evidence (GRADE)	What Happens
Schneiderian membrane perforation (intraoperative)	388 analysis units (11 trials reported; 9 included in the pooled estimate)	RR 0.63 (0.29 to 1.38) I^2^ = 9.2%	118 per 1000 with lateral 75 per 1000 with crestal (34 to 163) = 43 fewer per 1000 (84 fewer to 45 more)	⊕◯◯◯ VERY LOW a, b	The evidence is very uncertain about the effect of the crestal approach on Schneiderian membrane perforation.
Implant failure (12 months or longest follow-up)	223 analysis units (7 trials reported; 3 included in the pooled estimate)	RR 0.50 (0.10 to 2.61) I^2^ = 0%	36 per 1000 with lateral 18 per 1000 with crestal (3 to 93) = 18 fewer per 1000 (33 fewer to 57 more)	⊕◯◯◯ VERY LOW a, c	The evidence is very uncertain about the effect of the crestal approach on implant failure.
Peri-implant marginal bone loss (12 months)	119 analysis units (4 trials)	MD 0.08 mm lower (0.31 lower to 0.15 higher) I^2^ = 8.5%	Mean marginal bone loss with the crestal approach was 0.08 mm lower than with the lateral approach.	⊕⊕◯◯ LOW a, d	The crestal approach may result in little to no difference in peri-implant marginal bone loss at 12 months.
Vertical bone gain (12 months)	147 analysis units (4 trials)	MD 0.81 mm lower (1.94 lower to 0.33 higher) I^2^ = 67.2%	Mean vertical bone gain with the crestal approach was 0.81 mm lower than with the lateral approach.	⊕◯◯◯ VERY LOW a, e, f	The evidence is very uncertain about the effect of the crestal approach on vertical bone gain at 12 months.
Vertical bone gain (6 months)	147 analysis units (4 trials)	MD 1.07 mm lower (2.50 lower to 0.36 higher) I^2^ = 82.7%	Mean vertical bone gain with the crestal approach was 1.07 mm lower than with the lateral approach.	⊕◯◯◯ VERY LOW a, f, g	The evidence is very uncertain about the effect of the crestal approach on vertical bone gain at 6 months.
Postoperative pain (day of surgery to day 1)	136 participants (4 trials)	SMD 1.56 lower (3.31 lower to 0.20 higher) I^2^ = 82.1%	-	⊕◯◯◯ VERY LOW h, i, j, k	The evidence is very uncertain about the effect of the crestal approach on postoperative pain during the first day after surgery.

Patient or population: adults undergoing maxillary sinus floor augmentation with dental implant placement. Intervention: crestal (transalveolar) approach. Comparison: lateral window approach. Trials reporting zero events in both arms are shown in the forest plots but are excluded from the pooled estimate by the double-zero-event rule, so the number of trials contributing weight is smaller than the number reporting the outcome. Analysis units are patients, sinuses, sites or implants depending on how each trial reported the outcome; they are not interchangeable with the number of randomized participants. The unit-of-analysis limitation is stated in the Methods and in the Discussion. Footnotes: a. Downgraded one level for risk of bias: none of the trials contributing statistical weight to the primary pooled estimate was rated at low risk of bias for this outcome; the contributing judgments were rated as some concerns and/or high risk. b. Downgraded two levels for imprecision: only 35 events were observed, far below the optimal information size of approximately 300 events, and the confidence interval crosses both the appreciable-benefit and the appreciable-harm threshold (risk ratio 0.75 and 1.25). c. Downgraded two levels for imprecision: only 6 events were observed in the whole pool, and the confidence interval extends from a 90% relative reduction to a 161% relative increase in risk. d. Downgraded one level for imprecision because the confidence interval was not fully contained within a post hoc distribution-based minimal important difference of approximately 0.30 mm, calculated as half the pooled within-arm standard deviation of the four contributing trials; no validated anchor-based threshold was available. e. Downgraded one level for inconsistency: I^2^ = 67.2% (Q test *p* = 0.027); trial estimates ranged from −0.05 mm to −1.40 mm. f. Downgraded one level for imprecision: the confidence interval is compatible both with a clinically important difference in favor of the lateral approach and with no difference at all. g. Downgraded two levels for inconsistency: I^2^ = 82.7% (Q test *p* = 0.0006); the confidence intervals of the two most extreme trials do not overlap. h. Downgraded one level for risk of bias: all four contributing trials were rated at high risk of bias. Pain is an unblinded patient-reported outcome and no trial blinded participants. i. Downgraded two levels for inconsistency: I^2^ = 82.1% (Q test *p* = 0.0008). Leave-one-out analysis showed that a single trial (Lv 2022 [14]) accounted for all of the heterogeneity; without it the pooled estimate was SMD −1.07 (95% CI −1.61 to −0.53) with I^2^ = 0%. j. Downgraded one level for indirectness: the pool combines a 0–10 scale with 0–100 scales, timepoints ranging from the day of surgery to day 1 with one trial not stating its timepoint, and one set of values derived from a median and interquartile range. k. Downgraded one level for imprecision: 136 participants in total, and the confidence interval crosses the line of no effect while also including a very large benefit. Explanations: GRADE Working Group grades of evidence. High certainty (⊕⊕⊕⊕): we are very confident that the true effect lies close to that of the estimate of the effect. Moderate certainty (⊕⊕⊕◯): we are moderately confident in the effect estimate; the true effect is likely to be close to the estimate, but there is a possibility that it is substantially different. Low certainty (⊕⊕◯◯): our confidence in the effect estimate is limited; the true effect may be substantially different from the estimate. Very low certainty (⊕◯◯◯): we have very little confidence in the effect estimate; the true effect is likely to be substantially different from the estimate. The risk in the intervention group (and its 95% CI) is based on the assumed risk in the comparison group and the relative effect of the intervention (and its 95% CI). CI, confidence interval; MD, mean difference; RCT, randomized controlled trial; RR, risk ratio; SMD, standardized mean difference. Effect estimates are expressed as crestal versus lateral. A risk ratio below 1 favors the crestal approach, as does a mean difference below 0 for marginal bone loss and a standardized mean difference below 0 for postoperative pain. For vertical bone gain, where a larger value is the desirable outcome, a mean difference below 0 favors the lateral approach.

## Data Availability

All data analyzed during this review were derived from published reports and are included in this article and its Supplementary Materials. No individual participant data or unpublished study datasets were sought or obtained. The data extraction forms and analytic code are not publicly available.

## Supplementary Materials

The following supporting information can be downloaded at https://www.mdpi.com/article/10.3390/medicina62101973/s1, Figure S1: Outcome-level RoB 2 judgments; Table S1: Material deviations from and methodological additions to the original PROSPERO registration; Table S2: Search strategy of the online databases; Table S3: Reports excluded after full-text assessment, with reasons; Table S4: Risk of bias; Table S5: PRISMA 2020 checklist. References [9,25,31,39,40,41,42,43,44,45,46,47,48,49,50,51,52,53,54,55,56,57] are cited in the Supplementary Materials.

## Abbreviations

The following abbreviations are used in this manuscript:

CI	Confidence interval
GRADE	Grading of Recommendations Assessment, Development and Evaluation
MD	Mean difference
MID	Minimal important difference
PRISMA	Preferred Reporting Items for Systematic Reviews and Meta-Analyses
PROSPERO	International Prospective Register of Systematic Reviews
RCT	Randomized controlled trial
RoB 2	Cochrane Risk of Bias tool version 2
RR	Risk ratio
SMD	Standardized mean difference
VAS	Visual analogue scale
