# Supplementary material for: Crestal Versus Lateral Window Approaches for Maxillary Sinus Floor Augmentation: A Systematic Review and Meta-Analysis of Randomized Controlled Trials
Source: Medicina (Kaunas). 2026 Oct 12;62(10):1973. doi: 10.3390/medicina62101973 (PMC13706686; doi:10.3390/medicina62101973)
Supplement: Supplementary file 1 [file medicina-62-01973-s001.zip › medicina-4615855-supplementary.pdf]

Supplementary Table S1. Material deviations from and methodological additions to the original PROSPERO registration

No.	Domain	Original registration (12 July 2026)	Review as conducted	Reason and location in the report
**1**	Review scope and eligible study design	Both randomized and non-randomized study types were eligible. The registered question broadly addressed sinus augmentation techniques and biomaterials.	Eligibility was restricted to randomized and quasi-randomized controlled trials directly comparing crestal (transalveolar) and lateral window approaches. No eligible quasi-randomized trial was identified; all included trials were randomized. Grafting materials and biologic adjuncts were not treated as separate interventions.	The question was narrowed to one direct surgical comparison and to designs less susceptible to confounding. Reported in Methods (Protocol and eligibility criteria), the title, and Supplementary Table S1.
**2**	Risk-of-bias instrument	ROBINS-I was planned.	Cochrane RoB 2 was applied independently by two reviewers at the trial-outcome level (64 judgements across 13 trials).	This followed directly from the RCT-focused eligibility change: ROBINS-I is intended for non-randomized intervention studies, whereas RoB 2 is appropriate for randomized trials. Reported in Methods, Results, Figure 2, and Supplementary Table S4.
**3**	Certainty of evidence	Certainty of findings was not planned to be assessed.	GRADE was used for all six primary outcomes, with outcome-specific reasons for downgrading.	GRADE was added to provide an explicit assessment of confidence in each pooled estimate. Reported in Methods (Assessment of certainty of evidence), Results, and Table 2.
**4**	Outcome definitions and timepoints	Broad domains were listed: implant survival, implant success, increase in bone height, percentage of new bone formation, marginal bone loss, and complications. Specific analysis timepoints and effect measures were not defined.	Six primary outcomes were operationalized: implant failure; Schneiderian membrane perforation; marginal bone loss at 12 months; vertical bone gain at 6 and 12 months; and postoperative pain at the earliest timepoint within 24 hours. Implant success and new bone formation were not pooled because sufficiently comparable data were unavailable; other complications were summarized narratively.	The registered domains required clinically interpretable endpoints, timepoints, and effect measures for synthesis. Reported in Methods, Results, and Table 2. These operational definitions were not part of the original public registration.

No.	Domain	Original registration (12 July 2026)	Review as conducted	Reason and location in the report
**5**	Information sources	The information-sources field listed Embase, MEDLINE, PubMed, and SCIE; the original record also referred elsewhere to the Cochrane Database. Trial/study registers were selected as another method.	MEDLINE via PubMed, Embase, and the Cochrane Library were searched by a medical librarian. SCIE was not searched. One reviewer conducted a supplementary Google Scholar search; two reports were assessed and one was included. Registry records found through the searches were screened.	This row records the sources actually searched and resolves inconsistencies in the original record. Reported in the abstract, Methods (Information sources and search strategy), Results, Figure 1, and Supplementary Table S2.
**6**	Post hoc exclusion of two reports	No exclusion criterion based on research-ethics reporting, internal consistency, or data interpretability was registered.	Bensaha 2011 and Elsaid 2025 were excluded after full-text assessment because of post hoc concerns affecting data interpretability and applicability. Both reports were independently assessed by two reviewers, and the exclusion decisions were reached by consensus. Both reports were reintroduced in sensitivity analyses where usable outcome data were available.	The criterion was introduced after registration and is therefore explicitly labeled post hoc. The factual basis is reported in Supplementary Table S3; effects of reintroduction are reported in Results and Discussion. Neither sensitivity analysis changed a conclusion.
**7**	Unit-of-analysis rules	No rule was registered for pools mixing patient-, sinus-, site-, and implant-level data or for split-mouth and multi-arm trials.	Primary analyses preferentially used patient-, sinus-, or site-level data; all-units pools were sensitivity analyses. The restriction was not applied if it would leave two or fewer trials. The two eligible crestal arms in Temmerman 2017 were combined. Xhanari 2019 was entered as reported and assessed by exclusion sensitivity analyses.	The reporting units encountered during extraction required explicit rules to reduce unit-of-analysis error while retaining interpretable pools. Reported in Methods (Data synthesis and analysis) and in each affected Results subsection.

No.	Domain	Original registration (12 July 2026)	Review as conducted	Reason and location in the report
**8**	Meta-analytic procedures	The original public record did not specify software, estimators, sparse-event handling, conversion of medians and interquartile ranges, or detailed sensitivity-analysis procedures.	Analyses used R 4.4.2 and meta 8.5.0; RR, MD, or Hedges' g with 95% CIs; REML random-effects models except a Mantel–Haenszel fixed-effect model for sparse implant-failure data; Hartung–Knapp adjustment for continuous outcomes; and Wan et al. conversions when required. Double-zero trials were shown but received no weight.	These procedures were specified to match the outcome types and available data and are methodological additions rather than changes to a competing registered analysis. Reported in Methods (Data synthesis and analysis) and the relevant Results subsections.
**9**	Minimal important difference for marginal bone loss	No minimal important difference was registered; certainty of evidence was not originally planned.	GRADE imprecision for marginal bone loss was judged against a distribution-based threshold of approximately 0.30 mm, calculated as half the pooled within-arm standard deviation.	No validated anchor-based threshold was identified. The threshold was selected after the confidence interval was known and is therefore explicitly described as post hoc and data-dependent in Methods and Discussion; its effect is shown in Table 2.
**10**	Reporting-bias assessment	No method for assessing risk of bias due to missing results was registered.	A contour-enhanced funnel plot and Egger's regression test were used only when at least 10 trials contributed. This criterion was met only by the membrane-perforation all-units sensitivity pool (k = 10).	The threshold avoided unreliable formal testing in smaller pools. Reported in Methods (Assessment of reporting bias), Results (Assessment of small-study effects), and Figure 5.
**11**	Author contact and individual participant data	The original record stated that authors would be contacted for required unpublished information and that study datasets or individual participant data would be obtained.	No study author was contacted, and no study dataset or individual participant data was sought or obtained. Analyses used aggregate data in published reports.	The planned aggregate-data review was completed without these steps. This is now stated in Methods (Study selection and data extraction) and Supplementary Table S1.

The review was initially registered in PROSPERO on 12 July 2026 (CRD420261447905). After completion of the review, the PROSPERO record was amended to reflect the methods actually used. This table distinguishes the original registration from the completed review and states the reason for each material change or addition.

Note. “Original registration” refers to the PROSPERO record first registered on 12 July 2026, not the amended record. Administrative team updates are not listed because they did not alter eligibility, analysis, or interpretation. Abbreviations: CI, confidence interval; GRADE, Grading of Recommendations Assessment, Development and Evaluation; IPD, individual participant data; MD, mean difference; REML, restricted maximum likelihood; ROBINS-I, Risk Of Bias In Non-randomized Studies—of Interventions; RoB 2, Cochrane Risk of Bias tool version 2; RR, risk ratio; SCIE, Science Citation Index Expanded.

Supplementary Table S2. Search strategy of the online databases.

Database	Search	Search strategy
**Medline via PubMed**	#1	"Sinus Floor Augmentation"[Mesh]
	#2	"Sinus Floor Augmentation"[TW] OR "Sinus Floor Augmentations"[TW] OR "Maxillary Sinus Floor Augmentation"[TW] OR "Sinus Augmentation Therapy"[TW] OR "Sinus Augmentation Therapies"[TW] OR "maxillary sinus augmentation"[TW] OR "sinus augmentation"[TW] OR "sinus lift"[TW] OR "sinus lifting"[TW] OR "maxillary sinus floor elevation"[TW] OR "sinus elevation"[TW] OR "sinus graft"[TW] OR "sinus grafting"[TW] OR "sinus bone graft"[TW]
	#3	"Dental Implantation, Endosseous"[Mesh]
	#4	"Dental Implantation, Endosseous"[TW] OR "Endosseous Dental Implantation"[TW] OR "Endosseous Dental Implant Therapy"[TW] OR "Osseointegrated Dental Implantation"[TW] OR "Endosseous Implantation"[TW]
	#5<br>**Combine**	#1 OR #2 OR #3 OR #4<br>*P: maxillary sinus augmentation*
	#6<br>**Combine**	"crestal approach"[TW] OR "crestal implant approach"[TW] OR "crestal technique"[TW] OR "crestal sinus lift"[TW] OR "crestal sinus elevation"[TW] OR "transcrestal approach"[TW] OR "transcrestal technique"[TW] OR "transcrestal sinus lift"[TW] OR "transcrestal sinus floor elevation"[TW] OR "transcrestal sinus floor augmentation"[TW] OR "transalveolar approach"[TW] OR "transalveolar sinus elevation"[TW] OR "osteotome approach"[TW] OR "osteotome technique"[TW] OR "osteotome sinus floor elevation"[TW] OR "osteotome-mediated sinus elevation"[TW] OR "osteotome-mediated sinus floor elevation"[TW] OR "indirect sinus lift"[TW] OR "indirect sinus elevation"[TW] OR "closed sinus lift"[TW] OR "closed sinus elevation"[TW] OR "internal sinus lift"[TW] OR "Summers technique"[TW] OR "TSFE"[TW] OR "TSFA"[TW] OR "OSFE"[TW] OR "vertical approach"[TW]<br>*I: crestal approach*
	#7<br>**Combine**	"lateral approach"[TW] OR "lateral window"[TW] OR "lateral window approach"[TW] OR "lateral window technique"[TW] OR "lateral window sinus lift"[TW] OR "lateral window sinus elevation"[TW] OR "lateral technique"[TW] OR "lateral sinus floor elevation"[TW] OR "lateral sinus floor augmentation"[TW] OR "lateral maxillary sinus augmentation"[TW] OR "lateral antrostomy"[TW] OR "direct sinus lift"[TW] OR "direct sinus elevation"[TW] OR "open sinus lift"[TW] OR "open sinus elevation"[TW] OR "external sinus lift"[TW] OR "LSFE"[TW] OR "LSFA"[TW]<br>*C: lateral approach*

Database	Search	Search strategy
	#8<br>**Combine**	#5 AND #6 AND #7<br><br>*P AND I AND C*
Embase	#1	"sinus floor augmentation"/exp
	#2	"Sinus Floor Augmentation":ti,ab,kw OR "Sinus Floor Augmentations":ti,ab,kw OR "Maxillary Sinus Floor Augmentation":ti,ab,kw OR "Sinus Augmentation Therapy":ti,ab,kw OR "Sinus Augmentation Therapies":ti,ab,kw OR "maxillary sinus augmentation":ti,ab,kw OR "sinus augmentation":ti,ab,kw OR "sinus lift":ti,ab,kw OR "sinus lifting":ti,ab,kw OR "maxillary sinus floor elevation":ti,ab,kw OR "sinus elevation":ti,ab,kw OR "sinus graft":ti,ab,kw OR "sinus grafting":ti,ab,kw OR "sinus bone graft":ti,ab,kw
	**#3**	"tooth implantation"/exp
	#4	"Dental Implantation, Endosseous":ti,ab,kw OR "Endosseous Dental Implantation":ti,ab,kw OR "Endosseous Dental Implant Therapy":ti,ab,kw OR "Osseointegrated Dental Implantation":ti,ab,kw OR "Endosseous Implantation":ti,ab,kw
	#5<br><br>**Combine**	#1 OR #2 OR #3 OR #4<br><br>*P: maxillary sinus augmentation*
	#6	"crestal approach":ti,ab,kw OR "crestal implant approach":ti,ab,kw OR "crestal technique":ti,ab,kw OR "crestal sinus lift":ti,ab,kw OR "crestal sinus elevation":ti,ab,kw OR "transcrestal approach":ti,ab,kw OR "transcrestal technique":ti,ab,kw OR "transcrestal sinus lift":ti,ab,kw OR "transcrestal sinus floor elevation":ti,ab,kw OR "transcrestal sinus floor augmentation":ti,ab,kw OR "transalveolar approach":ti,ab,kw OR "transalveolar sinus elevation":ti,ab,kw OR "osteotome approach":ti,ab,kw OR "osteotome technique":ti,ab,kw OR "osteotome sinus floor elevation":ti,ab,kw OR "osteotome-mediated sinus elevation":ti,ab,kw OR "osteotome-mediated sinus floor elevation":ti,ab,kw OR "indirect sinus lift":ti,ab,kw OR "indirect sinus elevation":ti,ab,kw OR "closed sinus lift":ti,ab,kw OR "closed sinus elevation":ti,ab,kw OR "internal sinus lift":ti,ab,kw OR "Summers technique":ti,ab,kw OR "TSFE":ti,ab,kw OR "TSFA":ti,ab,kw OR "OSFE":ti,ab,kw OR "vertical approach":ti,ab,kw<br><br>*I: crestal approach*
	#7	"lateral approach":ti,ab,kw OR "lateral window":ti,ab,kw OR "lateral window approach":ti,ab,kw OR "lateral window technique":ti,ab,kw OR "lateral window sinus lift":ti,ab,kw OR "lateral window sinus elevation":ti,ab,kw OR "lateral technique":ti,ab,kw OR "lateral sinus floor elevation":ti,ab,kw OR "lateral sinus floor augmentation":ti,ab,kw OR "lateral maxillary sinus augmentation":ti,ab,kw OR "lateral antrostomy":ti,ab,kw OR "direct sinus lift":ti,ab,kw OR "direct sinus elevation":ti,ab,kw OR "open sinus lift":ti,ab,kw OR "open sinus elevation":ti,ab,kw OR "external sinus lift":ti,ab,kw OR

Database	Search	Search strategy
		"LSFE":ti,ab,kw OR "LSFA":ti,ab,kw<br>*C: lateral approach*
	#8<br>**Combine**	#5 AND #6 AND #7<br>*P AND I AND C*
**Cochrane Library**	#1	[mh "Sinus Floor Augmentation"]
	#2	"Sinus Floor Augmentation":ti,ab,kw OR "Sinus Floor Augmentations":ti,ab,kw OR "Maxillary Sinus Floor Augmentation":ti,ab,kw OR "Sinus Augmentation Therapy":ti,ab,kw OR "Sinus Augmentation Therapies":ti,ab,kw OR "maxillary sinus augmentation":ti,ab,kw OR "sinus augmentation":ti,ab,kw OR "sinus lift":ti,ab,kw OR "sinus lifting":ti,ab,kw OR "maxillary sinus floor elevation":ti,ab,kw OR "sinus elevation":ti,ab,kw OR "sinus graft":ti,ab,kw OR "sinus grafting":ti,ab,kw OR "sinus bone graft":ti,ab,kw
	#3	[mh "Dental Implantation, Endosseous"]
	#4	"Dental Implantation, Endosseous":ti,ab,kw OR "Endosseous Dental Implantation":ti,ab,kw OR "Endosseous Dental Implant Therapy":ti,ab,kw OR "Osseointegrated Dental Implantation":ti,ab,kw OR "Endosseous Implantation":ti,ab,kw
	#5<br>**Combine**	#1 OR #2 OR #3 OR #4<br>*P: maxillary sinus augmentation*
	#6<br>**Combine**	"crestal approach":ti,ab,kw OR "crestal implant approach":ti,ab,kw OR "crestal technique":ti,ab,kw OR "crestal sinus lift":ti,ab,kw OR "crestal sinus elevation":ti,ab,kw OR "transcrestal approach":ti,ab,kw OR "transcrestal technique":ti,ab,kw OR "transcrestal sinus lift":ti,ab,kw OR "transcrestal sinus floor elevation":ti,ab,kw OR "transcrestal sinus floor augmentation":ti,ab,kw OR "transalveolar approach":ti,ab,kw OR "transalveolar sinus elevation":ti,ab,kw OR "osteotome approach":ti,ab,kw OR "osteotome technique":ti,ab,kw OR "osteotome sinus floor elevation":ti,ab,kw OR "osteotome-mediated sinus elevation":ti,ab,kw OR "osteotome-mediated sinus floor elevation":ti,ab,kw OR "indirect sinus lift":ti,ab,kw OR "indirect sinus elevation":ti,ab,kw OR "closed sinus lift":ti,ab,kw OR "closed sinus elevation":ti,ab,kw OR "internal sinus lift":ti,ab,kw OR "Summers technique":ti,ab,kw OR "TSFE":ti,ab,kw OR "TSFA":ti,ab,kw OR "OSFE":ti,ab,kw OR "vertical approach":ti,ab,kw<br>*I: crestal approach*
	#7<br>**Combine**	"lateral approach":ti,ab,kw OR "lateral window":ti,ab,kw OR "lateral window approach":ti,ab,kw OR "lateral window technique":ti,ab,kw OR "lateral window sinus lift":ti,ab,kw OR "lateral window sinus elevation":ti,ab,kw OR "lateral technique":ti,ab,kw OR "lateral sinus floor elevation":ti,ab,kw OR "lateral sinus floor augmentation":ti,ab,kw OR "lateral maxillary sinus

Database	Search	Search strategy
		augmentation":ti,ab,kw OR "lateral antrostomy":ti,ab,kw OR "direct sinus lift":ti,ab,kw OR "direct sinus elevation":ti,ab,kw OR "open sinus lift":ti,ab,kw OR "open sinus elevation":ti,ab,kw OR "external sinus lift":ti,ab,kw OR "LSFE":ti,ab,kw OR "LSFA":ti,ab,kw<br><br>*C: lateral approach*
	**#8 Combine**	#5 AND #6 AND #7<br><br>*P AND I AND C*

Note. All database searches were run on 30 July 2026. Google Scholar was searched separately on the same day using the free-text query “sinus transcrestal lateral,” with the results limited to publication years from 2025 onward, followed by an author-based search. These supplementary searches were not archived, and the number of results reviewed was not recorded; they are described in the Methods.

Supplementary Table S3. Reports excluded after full-text assessment, with reasons.

Reason for exclusion	n	Records
Not a randomized or quasi-randomized controlled trial	14	1. Al-Almaie S, Kavarodi AM. Efficacy of dental implants placements between lateral and crestal sinus lift techniques: A split-mouth study. Open Dentistry Journal. 2019;13(1):346-357. *[39] — split-mouth comparison in 10 patients; the two techniques were not randomly allocated.*<br>2. Al-Almaie S, et al. A Split-Mouth Design Comparison for Lateral and Crestal Sinus Lift Techniques with Dental Implants Placements: Short Communication. Open Dent J. 2017;11:603-608. *[40] — short communication reporting the same split-mouth cohort as Al-Almaie and Kavarodi 2019 above; likewise not randomly allocated.*<br>3. D'Amato S, et al. [Maxillary sinus surgical lift. Summers' technique versus lateral surgical approach]. Minerva Stomatol. 2000;49(7-8):369-381. *[41] — retrospective comparison; article in Italian.*<br>4. Fatale V, et al. Histomorphometric Comparison of New Bone Formed After Maxillary Sinus Lift With Lateral and Crestal Approaches Using Periostal Mesenchymal Stem Cells and Beta-Tricalcium Phosphate: A Controlled Clinical Trial. J Craniofac Surg. 2022;33(5):1607-1613. *[42] — randomized allocation applied to the addition of mesenchymal stem cells only; the surgical approach was assigned by residual bone height (crestal 8-10 mm, lateral 4-8 mm).*<br>5. Kara MI, et al. Clinical evaluation of lateral and osteotome techniques for sinus floor elevation in the presence of an antral pseudocyst. Int J Oral Maxillofac Implants. 2012;27(5):1205-1210. *[43] — retrospective comparison.*<br>6. Kim SM, et al. Bone-added osteotome technique versus lateral approach for sinus floor elevation: a comparative radiographic study. Implant Dent. 2011;20(6):465-470. *[44] — non-randomized comparative radiographic study.*<br>7. Pal US, et al. Direct vs. indirect sinus lift procedure: A comparison. Natl J Maxillofac Surg. 2012;3(1):31-37. *[45] — non-randomized comparison.*<br>8. Palarie V, Zahalka M. Comparative study of transcrestal sinus lift versus lateral approach sinus augmentation and simultaneous insertion of bioactive calcium-phosphate coated dental implants: 12 years of follow-up. Journal of Clinical Periodontology. 2022;49:8-9. *[46] — conference abstract of a non-randomized prospective comparison.*<br>9. Park JH, et al. Longitudinal Study of Bone Height Change between Two Approaches for Sinus Floor Elevation. Medicina (Kaunas). 2023;59(6). *[47] — observational longitudinal study.*<br>10. Patel S, et al. Resonance Frequency Analysis of Sinus Augmentation by Osteotome Sinus Floor Elevation and Lateral Window Technique. J Oral Maxillofac Surg. 2015;73(10):1920-1925. *[48] — non-randomized comparative study.*<br>11. Rodoni LR, et al. Implants in the posterior maxilla: a comparative clinical and radiologic study. Int J Oral Maxillofac Implants. 2005;20(2):231-237. *[49] — observational comparative study.*<br>12. Tsai CF, et al. Comparison of 4 sinus augmentation techniques for implant placement with residual alveolar bone height ≤3 mm. Medicine (Baltimore). 2020;99(46):e23180. *[50] — retrospective comparison of four techniques.*<br>13. Uckan S, et al. Survival rates of implants inserted in the maxillary sinus area by internal or external approach. Implant Dentistry. 2011;20(6):476-479. *[51] — retrospective comparison.*<br>14. Zitzmann NU, Schärer P. Sinus elevation procedures in the resorbed posterior

Reason for exclusion	n	Records
		maxilla. Comparison of the crestal and lateral approaches. Oral Surg Oral Med Oral Pathol Oral Radiol Endod. 1998;85(1):8-17. *[52] — non-randomized prospective comparison.*
No direct comparison of the lateral versus the crestal approach	2	1. Ebenezer V, Ramalingam B. Efficacy of lateral antrostomy and crestal approach in maxillary sinus lift. European Journal of Molecular and Clinical Medicine. 2020;7(4):1574-1577. *[53] — narrative review; no comparative primary data are reported.*<br>2. ClinicalTrials.gov. Identifier NCT03496038. Transcrestal Lateral Sinus Floor Elevation in Implant Therapy. 2018. https://clinicaltrials.gov/show/NCT03496038 *[54] — both arms receive transcrestal sinus floor elevation; the randomized comparison is of leukocyte- and platelet-rich fibrin versus deproteinized bovine bone mineral as the grafting material.*
Trial registry record without published results	5	1. COMPARISON OF IMPLANT INSTALLATION USING THE TRANSCRESTAL APPROACH WITH OSSEODENSIFICATION OR THE LATERAL WINDOW TECHNIQUE WITH SINUS LIFT: A RANDOMIZED CONTROLLED TRIAL. 2022. *[55] — registry-derived record with no author or journal; the same trial as NCT06123221 below.*<br>2. ClinicalTrials.gov. Identifier NCT06123221. SINUS FLOOR WITH OSSEODENSIFICATION OR LATERAL WINDOW. 2022. https://clinicaltrials.gov/ct2/show/NCT06123221 *[55] — recruiting; no results posted and no full report identified.*<br>3. A 36 - Month Randomized Controlled Trial Comparing the Marginal Bone Changes Surrounding Dental Implants Placed with Open Versus Closed Sinus Elevation and Allogenic Bone Grafting. 2025. *[56] — registry-derived record with no author or journal; the same trial as NCT06811337 below.*<br>4. ClinicalTrials.gov. Identifier NCT06811337. Marginal Bone Changes Around Dental Implant Placed with Open Vs. Closed Sinus Elevation. 2025. https://clinicaltrials.gov/ct2/show/NCT06811337 *[56] — recorded as completed; no results posted and no full report identified.*<br>5. WHO International Clinical Trials Registry Platform. Identifier CTRI/2023/12/060904. Clinical outcomes of two different maxillary sinus floor augmentation procedures for Dental implant placement. 2023. https://trialsearch.who.int/Trial2.aspx?TrialID=CTRI/2023/12/060904 *[57] — recruiting; no results posted and no full report identified.*
Concerns affecting data interpretability and applicability	2	1. Bensaha T. Evaluation of the capability of a new water lift system to reduce the risk of Schneiderian membrane perforation during sinus elevation. Int J Oral Maxillofac Surg. 2011;40(8):815-820. *[31] — the sole author is stated to be the inventor of the device under evaluation; ethical approval is recorded as not required despite a randomized design; implant survival and failure are not reported. Identified through database searching.*<br>2. Elsaid MGAH, Aboelhasan MF, Shuman MAE, et al. Transcrestal sinus lift using osseodensification versus lateral window technique with simultaneous implant placement: a randomized clinical trial. Periodontal and Implant Research. 2025;9:article 16. *[25] — the Results report a highly significant increase in bone density in the crestal group while the Discussion states that no significant between-group difference was found; no numerical p value is reported; the trial was registered retrospectively. Identified through supplementary searching.*

Reason for exclusion	n	Records
**Total**	**23**	

Forty reports were assessed for eligibility across the two identification pathways (38 identified through database searching and 2 identified through supplementary searching), of which 23 were excluded. Reasons and counts correspond exactly to the eligibility-stage exclusion boxes of the PRISMA 2020 flow diagram (Figure 1): 22 reports from the database pathway and 1 report from the supplementary pathway. Records excluded at title and abstract screening (n = 165) and reports that could not be retrieved (n = 3) are not listed individually, in accordance with PRISMA 2020 item 16b, which asks for studies that might appear to meet the inclusion criteria.

Notes

The two reports excluded for concerns affecting data interpretability and applicability were identified through different pathways, which is why Figure 1 shows one such report in each of the two exclusion boxes. Both decisions were taken after full-text assessment and after the protocol had been registered, and are reported as a post hoc deviation in the Methods.

The five trial registry records correspond to three distinct trials: two records describe NCT06123221, two describe NCT06811337, and one describes CTRI/2023/12/060904. Each was retrieved separately by the search and is therefore counted separately, in accordance with PRISMA 2020 reporting of reports rather than studies.

Both Al-Almaie reports describe the same split-mouth cohort. Both are listed under the study design criterion because neither used random allocation, which is the first eligibility criterion applied; the duplication is noted in the annotation to the second report.

Reasons and counts in this table match the eligibility-stage exclusion boxes of Figure 1 exactly.

Citations for excluded reports were transcribed from the screening records exported from the reference manager. Journal titles and author lists are presented as recorded there.

Supplementary Table S4. Risk of bias.

Study	Outcome	D1: Randomization process	D2: Deviations from the intended interventions	D3: Missing outcome data	D4: Measurement of the outcome	D5: Selection of the reported result	Overall Bias
Wittmers (2026)	Implant failure	Some concerns	High risk	Some concerns	Some concerns	High risk	High risk
Wittmers (2026)	Membrane perforation	Some concerns	Some concerns	Low risk	Some concerns	High risk	High risk
Wittmers (2026)	Vertical bone gain	Some concerns	High risk	High risk	Some concerns	High risk	High risk
Mattar (2026)	Implant failure	High risk	Some concerns	Some concerns	Low risk	Some concerns	High risk
Mattar (2026)	Membrane perforation	High risk	Some concerns	Some concerns	Some concerns	Some concerns	High risk
Mattar (2026)	Marginal bone loss	High risk	Some concerns	Some concerns	Low risk	Some concerns	High risk
Mattar (2026)	Vertical bone gain	High risk	Some concerns	Some concerns	Some concerns	Some concerns	High risk
Fettouh (2026)	Implant failure	Low risk	Low risk	Low risk	Low risk	Low risk	Low risk
Fettouh (2026)	Membrane perforation	Low risk	Low risk	Low risk	High risk	Low risk	High risk
Fettouh (2026)	Marginal bone loss	Low risk	Low risk	Low risk	Some concerns	Low risk	Some concerns
Fettouh (2026)	Vertical bone gain	Low risk	Low risk	Low risk	Some concerns	Low risk	Some concerns
Fettouh (2026)	Postoperative pain (VAS)	Low risk	Low risk	Low risk	High risk	Low risk	High risk
Cheng (2026)	Implant failure	Some concerns	Low risk	Low risk	Some concerns	Some concerns	Some concerns
Cheng (2026)	Membrane perforation	Some concerns	Low risk	Low risk	Some concerns	Some concerns	Some concerns
Cheng (2026)	Vertical bone gain	Some concerns	Low risk	Low risk	Some concerns	Some concerns	Some concerns
Cheng (2026)	Peri-implant disease (mucositis / peri-implantitis)	Some concerns	Low risk	Low risk	Some concerns	Some concerns	Some concerns

Study	Outcome	D1: Randomization process	D2: Deviations from the intended interventions	D3: Missing outcome data	D4: Measurement of the outcome	D5: Selection of the reported result	Overall Bias
Cheng (2026)	Postoperative pain (VAS)	Some concerns	Low risk	Low risk	High risk	Some concerns	High risk
Cheng (2026)	Surgical time	Some concerns	Low risk	Low risk	Some concerns	Some concerns	Some concerns
Cheng (2026)	Hospital Anxiety and Depression Scale (behavioural cognition)	Some concerns	Low risk	Low risk	High risk	Some concerns	High risk
Gaspar (2024)	Implant failure	Low risk	Low risk	Low risk	Some concerns	Low risk	Some concerns
Gaspar (2024)	Membrane perforation	Low risk	Low risk	Low risk	High risk	Low risk	High risk
Gaspar (2024)	Postoperative infection	Low risk	Low risk	Low risk	Some concerns	Low risk	Some concerns
Gaspar (2024)	Postoperative pain (VAS)	Low risk	Some concerns	Low risk	High risk	Low risk	High risk
Gaspar (2024)	Swelling	Low risk	Some concerns	Low risk	High risk	Low risk	High risk
Gaspar (2024)	Surgical time	Low risk	Low risk	Low risk	Some concerns	Low risk	Some concerns
Farina (2018-2023)	Implant failure	Low risk	Low risk	Low risk	Low risk	Low risk	Low risk
Farina (2018-2023)	Membrane perforation	Low risk	Low risk	Low risk	Some concerns	Low risk	Some concerns
Farina (2018-2023)	Marginal bone loss	Low risk	Low risk	Some concerns	Some concerns	Some concerns	Some concerns
Farina (2018-2023)	Postoperative pain (VAS)	Low risk	Low risk	Low risk	High risk	Low risk	High risk
Farina (2018-2023)	Swelling	Low risk	Low risk	Low risk	High risk	Low risk	High risk
Farina (2018-2023)	Surgical time	Low risk	Low risk	Low risk	Low risk	Low risk	Low risk
Lv (2022)	Implant failure	Some concerns	Low risk	Low risk	Low risk	Low risk	Some concerns

Study	Outcome	D1: Randomization process	D2: Deviations from the intended interventions	D3: Missing outcome data	D4: Measurement of the outcome	D5: Selection of the reported result	Overall Bias
Lv (2022)	Membrane perforation	Some concerns	Low risk	Low risk	Low risk	Low risk	Some concerns
Lv (2022)	Marginal bone loss	Some concerns	Low risk	Some concerns	Some concerns	Some concerns	Some concerns
Lv (2022)	Vertical bone gain	Some concerns	Low risk	Low risk	Some concerns	Low risk	Some concerns
Lv (2022)	Postoperative pain (VAS)	Some concerns	Low risk	Low risk	High risk	Low risk	High risk
Lv (2022)	Swelling	Some concerns	Low risk	Low risk	High risk	Low risk	High risk
Zhou (2021)	Implant failure	Some concerns	Low risk	Low risk	Low risk	Low risk	Some concerns
Zhou (2021)	Membrane perforation	Some concerns	Low risk	Low risk	Low risk	Some concerns	Some concerns
Zhou (2021)	Marginal bone loss	Some concerns	Low risk	Some concerns	Some concerns	Some concerns	Some concerns
Zhou (2021)	Vertical bone gain	Some concerns	Low risk	Low risk	Some concerns	Low risk	Some concerns
Bacevic (2021)	Implant failure	Some concerns	Low risk	Some concerns	Low risk	Low risk	Some concerns
Bacevic (2021)	Membrane perforation	Some concerns	Low risk	Low risk	High risk	Low risk	High risk
Bacevic (2021)	Vertical bone gain	Some concerns	Low risk	Some concerns	Some concerns	Some concerns	Some concerns
Bacevic (2021)	Patient satisfaction (VAS)	Some concerns	Low risk	Low risk	High risk	Low risk	High risk
Bacevic (2021)	Swelling	Some concerns	Low risk	Low risk	High risk	Low risk	High risk
Bacevic (2021)	Surgical time	Some concerns	Low risk	Low risk	Low risk	Low risk	Some concerns
Cannizzaro (2013/2020)	Implant failure	Low risk	Low risk	Low risk	Some concerns	Low risk	Some concerns
Cannizzaro (2013/2020)	Membrane perforation	Low risk	Low risk	Low risk	Some concerns	Low risk	Some concerns
Cannizzaro (2013/2020)	Marginal bone loss	Low risk	Low risk	Low risk	High risk	Low risk	High risk

Study	Outcome	D1: Randomization process	D2: Deviations from the intended interventions	D3: Missing outcome data	D4: Measurement of the outcome	D5: Selection of the reported result	Overall Bias
Cannizzaro (2013/2020)	Postoperative infection	Low risk	Low risk	Low risk	Some concerns	Low risk	Some concerns
Yu (2017)	Implant failure	Some concerns	Low risk	Low risk	Low risk	Low risk	Some concerns
Yu (2017)	Membrane perforation	Some concerns	Low risk	Low risk	Some concerns	Low risk	Some concerns
Yu (2017)	Marginal bone loss	Some concerns	Low risk	Some concerns	Some concerns	Some concerns	Some concerns
Yu (2017)	Vertical bone gain	Some concerns	Low risk	Low risk	Some concerns	Low risk	Some concerns
Yu (2017)	Postoperative infection	Some concerns	Low risk	Low risk	Low risk	Low risk	Some concerns
Yu (2017)	Postoperative discomfort (dichotomous)	Some concerns	Low risk	Some concerns	High risk	High risk	High risk
Temmerman (2017)	Membrane perforation	Some concerns	Low risk	Low risk	High risk	Some concerns	High risk
Temmerman (2017)	Vertical bone gain	Some concerns	Low risk	Some concerns	Low risk	Some concerns	Some concerns
Temmerman (2017)	Postoperative pain (VAS)	Some concerns	Low risk	Low risk	High risk	Some concerns	High risk
Temmerman (2017)	Swelling	Some concerns	Low risk	Low risk	High risk	Some concerns	High risk
Xhanari (2019)	Implant failure	Low risk	Some concerns	Low risk	Some concerns	Some concerns	Some concerns
Xhanari (2019)	Marginal bone loss	Low risk	Some concerns	Some concerns	Some concerns	Some concerns	Some concerns
Xhanari (2019)	Surgical time	Low risk	Some concerns	Low risk	Some concerns	Some concerns	Some concerns

Supplementary Figure S1. Outcome-level RoB 2 judgments. Traffic-light plot showing domain-level and overall RoB 2 judgments for each trial–outcome pair.

Study ID	Outcome	D1	D2	D3	D4	D5	Overall
Wittmers 2026	Implant failure	!	-	!	!	-	-
Wittmers 2026	Membrane perforation	!	!	+	!	-	-
Wittmers 2026	Vertical bone gain	!	-	-	!	-	-
Mattar 2026	Implant failure	-	!	!	+	!	-
Mattar 2026	Membrane perforation	-	!	!	!	!	-
Mattar 2026	Marginal bone loss	-	!	!	+	!	-
Mattar 2026	Vertical bone gain	-	!	!	!	!	-
Fettouh 2026	Implant failure	+	+	+	+	+	+
Fettouh 2026	Membrane perforation	+	+	+	-	+	-
Fettouh 2026	Marginal bone loss	+	+	+	!	+	!
Fettouh 2026	Vertical bone gain	+	+	+	!	+	!
Fettouh 2026	Postoperative pain (VAS)	+	+	+	-	+	-
Cheng 2026	Implant failure	!	+	+	!	!	!
Cheng 2026	Membrane perforation	!	+	+	!	!	!
Cheng 2026	Vertical bone gain	!	+	+	!	!	!
Cheng 2026	Peri-implant disease (mucositis / peri-implantitis)	!	+	+	!	!	!
Cheng 2026	Postoperative pain (VAS)	!	+	+	-	!	-
Cheng 2026	Surgical time	!	+	+	!	!	!
Cheng 2026	Hospital Anxiety and Depression Scale (behavioural cognition)	!	+	+	-	!	-
Gaspar 2024	Implant failure	+	+	+	!	+	!
Gaspar 2024	Membrane perforation	+	+	+	-	+	-
Gaspar 2024	Postoperative infection	+	+	+	!	+	!
Gaspar 2024	Postoperative pain (VAS)	+	!	+	-	+	-
Gaspar 2024	Swelling	+	!	+	-	+	-
Gaspar 2024	Surgical time	+	+	+	!	+	!
Farina 2018-2023	Implant failure	+	+	+	+	+	+
Farina 2018-2023	Membrane perforation	+	+	+	!	+	!
Farina 2018-2023	Marginal bone loss	+	+	!	!	!	!
Farina 2018-2023	Postoperative pain (VAS)	+	+	+	-	+	-
Farina 2018-2023	Swelling	+	+	+	-	+	-
Farina 2018-2023	Surgical time	+	+	+	+	+	+
Lv 2022	Implant failure	!	+	+	+	+	!
Lv 2022	Membrane perforation	!	+	+	+	+	!
Lv 2022	Marginal bone loss	!	+	!	!	!	!
Lv 2022	Vertical bone gain	!	+	+	!	+	!
Lv 2022	Postoperative pain (VAS)	!	+	+	-	+	-
Lv 2022	Swelling	!	+	+	-	+	-
Zhou 2021	Implant failure	!	+	+	+	+	!
Zhou 2021	Membrane perforation	!	+	+	+	!	!
Zhou 2021	Marginal bone loss	!	+	!	!	!	!
Zhou 2021	Vertical bone gain	!	+	+	!	+	!
Bacevic 2021	Implant failure	!	+	!	+	+	!
Bacevic 2021	Membrane perforation	!	+	+	-	+	-
Bacevic 2021	Vertical bone gain	!	+	!	!	!	!
Bacevic 2021	Patient satisfaction (VAS)	!	+	+	-	+	-
Bacevic 2021	Swelling	!	+	+	-	+	-
Bacevic 2021	Surgical time	!	+	+	+	+	!
Cannizzaro 2013/2020	Implant failure	+	+	+	!	+	!
Cannizzaro 2013/2020	Membrane perforation	+	+	+	!	+	!
Cannizzaro 2013/2020	Marginal bone loss	+	+	+	-	+	-
Cannizzaro 2013/2020	Postoperative infection	+	+	+	!	+	!
Yu 2017	Implant failure	!	+	+	+	+	!
Yu 2017	Membrane perforation	!	+	+	!	+	!
Yu 2017	Marginal bone loss	!	+	!	!	!	!
Yu 2017	Vertical bone gain	!	+	+	!	+	!
Yu 2017	Postoperative infection	!	+	+	+	+	!
Yu 2017	Postoperative discomfort (dichotomous)	!	+	!	-	-	-
Temmerman 2017	Membrane perforation	!	+	+	-	!	-
Temmerman 2017	Vertical bone gain	!	+	!	+	!	!
Temmerman 2017	Postoperative pain (VAS)	!	+	+	-	!	-
Temmerman 2017	Swelling	!	+	+	-	!	-
Xhanari 2019	Implant failure	+	!	+	!	!	!
Xhanari 2019	Marginal bone loss	+	!	!	!	!	!
Xhanari 2019	Surgical time	+	!	+	!	!	!

+ Low risk
! Some concerns
- High risk

D1 Randomisation process
D2 Deviations from the intended interventions
D3 Missing outcome data
D4 Measurement of the outcome
D5 Selection of the reported result

Supplementary Table S5. PRISMA 2020 checklist.

Section and Topic	Item #	Checklist item	Location where item is reported
**TITLE**			
Title	1	Identify the report as a systematic review.	Title
**ABSTRACT**			
Abstract	2	See the PRISMA 2020 for Abstracts checklist.	Abstract
**INTRODUCTION**			
Rationale	3	Describe the rationale for the review in the context of existing knowledge.	Introduction (paragraphs 1–2)
Objectives	4	Provide an explicit statement of the objective(s) or question(s) the review addresses.	Introduction (final paragraph)
**METHODS**			
Eligibility criteria	5	Specify the inclusion and exclusion criteria for the review and how studies were grouped for the syntheses.	Materials and methods — Protocol and eligibility criteria
Information sources	6	Specify all databases, registers, websites, organisations, reference lists and other sources searched or consulted to identify studies. Specify the date when each source was last searched or consulted.	Materials and methods — Information sources and search strategy; Supplementary Table S2
Search strategy	7	Present the full search strategies for all databases, registers and websites, including any filters and limits used.	Materials and methods — Information sources and search strategy; Supplementary Table S2
Selection process	8	Specify the methods used to decide whether a study met the inclusion criteria of the review, including how many reviewers screened each record and each report retrieved, whether they worked independently, and if applicable, details of automation tools used in the process.	Materials and methods — Study selection and data extraction
Data collection process	9	Specify the methods used to collect data from reports, including how many reviewers collected data from each report, whether they worked independently, any processes for obtaining or confirming data from study investigators, and if applicable, details of automation tools used in the process.	Materials and methods — Study selection and data extraction
Data items	10a	List and define all outcomes for which data were sought. Specify whether all results that were compatible with each outcome domain in each study were sought (e.g. for all measures, time points, analyses), and if not, the methods used to decide which results to collect.	Materials and methods — Protocol and eligibility criteria; Data synthesis and analysis
Data items	10b	List and define all other variables for which data were sought (e.g. participant and intervention characteristics, funding sources). Describe any assumptions made about any missing or unclear information.	Materials and methods — Study selection and data extraction; Table 1
Study risk of bias assessment	11	Specify the methods used to assess risk of bias in the included studies, including details of the tool(s) used, how many reviewers assessed each study and whether they worked independently, and if applicable, details of automation tools used in the process.	Materials and methods — Risk of bias assessment
Effect measures	12	Specify for each outcome the effect measure(s) (e.g. risk ratio, mean difference) used in the synthesis or presentation of results.	Materials and methods — Data synthesis and analysis
Synthesis methods	13a	Describe the processes used to decide which studies were eligible for each synthesis (e.g. tabulating the study intervention characteristics and comparing against the planned groups for each synthesis (item #5)).	Materials and methods — Data synthesis and analysis; Results — Meta-analysis
Synthesis methods	13b	Describe any methods required to prepare the data for presentation or synthesis, such as handling of missing summary statistics, or data conversions.	Materials and methods — Data synthesis and analysis
Synthesis methods	13c	Describe any methods used to tabulate or visually display results of individual studies and syntheses.	Materials and methods — Data synthesis and analysis; Figures 2–5; Tables 1 and 2
Synthesis methods	13d	Describe any methods used to synthesize results and provide a rationale for the choice(s). If meta-analysis was performed, describe the model(s), method(s) to identify the presence and extent of statistical heterogeneity, and software package(s) used.	Materials and methods — Data synthesis and analysis

Section and Topic	Item #	Checklist item	Location where item is reported
Synthesis methods	13e	Describe any methods used to explore possible causes of heterogeneity among study results (e.g. subgroup analysis, meta-regression).	Section 2.5. No formal subgroup analysis or meta-regression was conducted. Exploratory sensitivity and leave-one-out analyses are described under item 13f.
Synthesis methods	13f	Describe any sensitivity analyses conducted to assess robustness of the synthesized results.	Materials and methods — Data synthesis and analysis
Reporting bias assessment	14	Describe any methods used to assess risk of bias due to missing results in a synthesis (arising from reporting biases).	Materials and methods — Assessment of reporting bias
Certainty assessment	15	Describe any methods used to assess certainty (or confidence) in the body of evidence for an outcome.	Materials and methods — Assessment of certainty of evidence
**RESULTS**			
Study selection	16a	Describe the results of the search and selection process, from the number of records identified in the search to the number of studies included in the review, ideally using a flow diagram.	Section 3.1, Search Results and Study Characteristics; Figure 1.
Study selection	16b	Cite studies that might appear to meet the inclusion criteria, but which were excluded, and explain why they were excluded.	Section 3.1, Search Results and Study Characteristics; Supplementary Table S3.
Study characteristics	17	Cite each included study and present its characteristics.	Section 3.1, Search Results and Study Characteristics; Table 1.
Risk of bias in studies	18	Present assessments of risk of bias for each included study.	Section 3.2, Risk of Bias in Included Studies; Figure 2; Supplementary Figure S1; Supplementary Table S4.
Results of individual studies	19	For all outcomes, present, for each study: (a) summary statistics for each group (where appropriate) and (b) an effect estimate and its precision (e.g. confidence/credible interval), ideally using structured tables or plots.	Sections 3.4–3.9; Figures 3 and 4; Table 1.
Results of syntheses	20a	For each synthesis, briefly summarise the characteristics and risk of bias among contributing studies.	Sections 3.2–3.8 and 3.11; Tables 1 and 2; Supplementary Table S4.
Results of syntheses	20b	Present results of all statistical syntheses conducted. If meta-analysis was done, present for each the summary estimate and its precision (e.g. confidence/credible interval) and measures of statistical heterogeneity. If comparing groups, describe the direction of the effect.	Sections 3.4–3.8; Figures 3 and 4; Table 2.
Results of syntheses	20c	Present results of all investigations of possible causes of heterogeneity among study results.	Not applicable; no formal subgroup analysis or meta-regression was conducted.
Results of syntheses	20d	Present results of all sensitivity analyses conducted to assess the robustness of the synthesized results.	Sections 3.4–3.8, including the outcome-specific sensitivity analyses.
Reporting biases	21	Present assessments of risk of bias due to missing results (arising from reporting biases) for each synthesis assessed.	Results — Assessment of small-study effects; Figure 5
Certainty of evidence	22	Present assessments of certainty (or confidence) in the body of evidence for each outcome assessed.	Results — Certainty of evidence; Table 2
**DISCUSSION**			
Discussion	23a	Provide a general interpretation of the results in the context of other evidence.	Discussion (paragraphs 1–4)
Discussion	23b	Discuss any limitations of the evidence included in the review.	Discussion (limitations paragraph)
Discussion	23c	Discuss any limitations of the review processes used.	Discussion (limitations paragraph)
Discussion	23d	Discuss implications of the results for practice, policy, and future research.	Discussion (final paragraph); Conclusions
**OTHER INFORMATION**			
Registration and protocol	24a	Provide registration information for the review, including register name and registration number, or state that the review was not registered.	Materials and methods — Protocol and eligibility criteria (PROSPERO, CRD420261447905)

Section and Topic	Item #	Checklist item	Location where item is reported
Registration and protocol	24b	Indicate where the review protocol can be accessed, or state that a protocol was not prepared.	Materials and methods — Protocol and eligibility criteria (PROSPERO CRD420261447905)
Registration and protocol	24c	Describe and explain any amendments to information provided at registration or in the protocol.	Materials and methods — Protocol and eligibility criteria; Supplementary Table S1
Support	25	Describe sources of financial or non-financial support for the review, and the role of the funders or sponsors in the review.	Funding; Author Contributions (Search Strategy Design and Execution); Section 2.2.
Competing interests	26	Declare any competing interests of review authors.	Conflicts of Interest
Availability of data, code and other materials	27	Report which of the following are publicly available and where they can be found: template data collection forms; data extracted from included studies; data used for all analyses; analytic code; any other materials used in the review.	Data Availability Statement; Table 1; Figures 3–5; Supplementary Table S4. All analysed data were derived from published reports and are included in the article and Supplementary Information. Data extraction forms and analytic code are not publicly available.

From: Page MJ, McKenzie JE, Bossuyt PM, Boutron I, Hoffmann TC, Mulrow CD, et al. The PRISMA 2020 statement: an updated guideline for reporting systematic reviews. *Syst. Rev.* **2021**, *10*, 89. doi: 10.1186/s13643-021-01626-4. This work is licensed under CC BY 4.0. To view a copy of this license, visit https://creativecommons.org/licenses/by/4.0/
